# Long non-coding RNA-mediated stress adaptation and immune remodeling in non-small cell lung cancer: molecular mechanisms and therapeutic implications

**DOI:** 10.3389/fimmu.2026.1982129

**Published:** 2026-10-22

**Authors:** Fuyao Shang, Yiqiu Zhang, Qingyuan Zhou, Mengxiang Zhu, Fengyuan He, Mengxiang Li, Tiesuo Zhao, Sheng Guo, Jiateng Zhong, Zhijun Yang, Fei Tu

**Affiliations:** 1 School of Forensic Medicine, Henan Medical University, Xinxiang, Henan, China; 2 Xinxiang Engineering Technology Research Center of Immune Checkpoint Drug for Liver-Intestinal Tumors, Henan Medical University, Xinxiang, Henan, China; 3 School of Basic Medical Sciences, Henan Medical University, Xinxiang, Henan, China

**Keywords:** competing endogenous RNA, ferroptosis, long non-coding RNA, metabolic reprogramming, non-small cell lung cancer, stress-sensing lncRNA, therapeutic resistance, tumor microenvironment

## Abstract

Non-small cell lung cancer (NSCLC) remains the predominant subtype of lung cancer and a leading cause of cancer-related mortality worldwide. Therapeutic failure is largely driven by intratumoral heterogeneity, dynamic tumor microenvironmental stresses, and adaptive drug resistance. Unlike previous reviews focusing on isolated lncRNA–target axes, this review proposes an integrated conceptual framework emphasizing that four major classes of tumor-associated stresses are highly interconnected through a cross-stress compensatory network. Within this framework, lncRNAs serve as central yet functionally stratified regulatory nodes bridging stress sensing and adaptive reprogramming. We propose a functional continuum rather than a fixed binary classification: stress-sensing and downstream effector lncRNAs represent relative positions along a stress-input-to-adaptive-output axis, and the same lncRNA may switch or combine these roles depending on cell type, genetic background, stress intensity, and treatment context. We systematically illustrate how stress-responsive lncRNAs reshape metabolic reprogramming, redox homeostasis, ferroptosis susceptibility, immune evasion, autophagy, DNA-damage repair, and therapeutic resistance. We further show that these four stress conditions do not operate in isolation; rather, metabolites, ROS, hypoxia-responsive signaling, and intercellular communication generate reciprocal feedback and cross-compensation that together sustain tumor survival under sustained pressure. Beyond mechanistic integration, we discuss the translational prospects of stress-related lncRNAs as diagnostic and prognostic biomarkers, therapeutic targets, and mediators for mechanism-based combination therapies. However, it is critical to note that no lncRNA-targeting therapeutic agent has yet entered clinical trials for NSCLC, and the path from bench to bedside remains constrained by delivery challenges, model fidelity limitations, and insufficient standardization of clinical assays. Comprehensive dissection of lncRNA-mediated stress-adaptation networks will deepen our understanding of NSCLC pathogenesis and facilitate the development of precision medicine strategies.

## 1 Introduction

Lung cancer remains the most frequently diagnosed malignancy and the leading cause of cancer-related mortality worldwide. According to the GLOBOCAN 2024 database, approximately 2.637 million new lung cancer cases and 1.862 million lung cancer-related deaths were estimated globally in 2024, representing a substantial public health burden (1). On the basis of histopathological and molecular characteristics, lung cancer is mainly classified into non-small cell lung cancer (NSCLC, approximately 85%) and small cell lung cancer (SCLC, approximately 15%), which exhibit distinct oncogenic alterations and tumor microenvironmental features (2). This review focuses on NSCLC, the predominant clinical subtype of lung cancer. Although EGFR/ALK tyrosine kinase inhibitors, immune checkpoint inhibitors, and platinum-based chemotherapy have significantly expanded therapeutic options for advanced NSCLC, intratumoral heterogeneity and multiple microenvironmental stresses remain major determinants of treatment failure and limited therapeutic efficacy. The 5-year survival rate of patients with advanced NSCLC remains below 25% (3).

Solid tumors are continuously exposed to four major stress conditions: metabolic stress induced by nutrient deprivation, oxidative stress and ferroptotic stress driven by lipid peroxidation, immunosuppressive microenvironmental stress, and therapy-induced stress caused by chemotherapy, radiotherapy, and targeted treatment. These stress states are highly interconnected rather than independent processes. Through metabolic remodeling, redox regulation, immune modulation, and adaptive signaling, they in concert promote tumor proliferation, epithelial–mesenchymal transition (EMT), maintenance of cancer stemness, immunosuppressive niche formation, and multidrug resistance (4–6). With this in mind, systematic investigation of stress adaptation networks is essential for identifying reliable biomarkers and novel therapeutic targets in NSCLC.

NSCLC is not a single disease. LUAD and LUSC, as well as genetically defined subsets such as EGFR-mutant, KRAS-mutant, STK11/LKB1-deficient, and KEAP1/NRF2-altered tumors, differ substantially in metabolic dependencies, redox regulation, ferroptosis sensitivity, immune context, and therapeutic response. Consequently, mechanisms described in one histological or genomic context should not be generalized to all NSCLC without validation across relevant subtypes and mutational backgrounds.

Intratumoral heterogeneity further complicates this picture. Different subclones and spatially separated tumor regions experience distinct gradients of oxygen, nutrients, lactate, ROS, immune cells, and therapeutic exposure. A single-biopsy measurement may therefore reflect the sampled region rather than a tumor-wide dependency, and static single-time-point biomarkers may not capture dynamic stress adaptation. These limitations motivate multi-region sampling, spatial and single-cell approaches, and longitudinal liquid biopsy, as discussed later.

Long non-coding RNAs (lncRNAs) are RNA transcripts longer than 200 nucleotides with limited protein-coding potential. Initially regarded as transcriptional by-products, lncRNAs have been increasingly recognized as functional regulatory molecules through advances in high-throughput sequencing and functional validation studies (7). Building upon their interactions with RNA, proteins, and DNA, lncRNAs are generally classified into four functional categories: competing endogenous RNA (ceRNA) sponges, protein scaffolds, chromatin-guiding molecules, and stress-responsive signaling molecules (8–10). Classical studies categorized lncRNA functions into Signal, Decoy, Guide, and Scaffold models (10). These four functional categories are detailed in Chapter 2.

Representative studies have demonstrated the diverse regulatory modes of lncRNAs in NSCLC. MALAT1 functions as a ceRNA by interacting with miR-145 and regulating NSCLC cell proliferation, highlighting the role of cytoplasmic lncRNAs in post-transcriptional regulation (11). LINK-A acts as a protein scaffold to stabilize HIF-1α and facilitate hypoxic adaptation, demonstrating the involvement of lncRNA-mediated protein complex regulation in tumor survival (12). HOTTIP regulates glycolysis-associated gene expression through chromatin-associated mechanisms and contributes to metabolic adaptation under hypoxic conditions (13). GAS5 responds to therapeutic stimulation and participates in chemotherapy resistance, indicating that lncRNAs also serve as stress-responsive regulators under treatment selection pressure (14).

Accumulating evidence further demonstrates that stress-associated lncRNAs are involved in multiple adaptive processes in NSCLC. Under metabolic stress, LINC00662 responds to nutrient deprivation and regulates metabolic remodeling through epigenetic mechanisms, linking environmental changes with transcriptional plasticity (15). During oxidative stress, SLC7A11AR activates the SLC7A11 pathway through ceRNA regulation and suppresses ferroptosis by maintaining redox homeostasis (16). Under hypoxic and immunosuppressive conditions, LINC00301 enhances HIF-1α signaling, promotes EMT, and contributes to immune microenvironment remodeling through increased regulatory T-cell infiltration (17). During therapeutic stress, SNHG15 and TRIDENT regulate drug efflux and DNA damage repair pathways, respectively, thereby promoting acquired resistance to anticancer therapies (18, 19). Viewed together, these findings indicate that lncRNAs function as central regulators connecting environmental stress signals with adaptive tumor phenotypes.

Beyond their mechanistic functions, stress-associated lncRNAs exhibit considerable clinical translation potential. Their abnormal expression patterns are closely associated with tumor progression, prognosis, and therapeutic response, supporting further investigation as candidate diagnostic and prognostic biomarkers (20). lncRNAs exhibit relatively high stability in tumor tissues and extracellular vesicles, particularly exosomal lncRNAs, which are detectable in peripheral blood and other body fluids. These circulating molecules reflect tumor biological characteristics and provide potential tools for minimally invasive liquid biopsy and dynamic disease monitoring (21). Furthermore, targeting stress-associated lncRNAs represents a promising therapeutic strategy. Modulating lncRNA-mediated stress adaptation pathways restore drug sensitivity and provide novel molecular targets for combination therapies in NSCLC (22).

Several existing reviews have delineated the roles of lncRNAs in NSCLC from perspectives including autophagy regulation, immune modulation, and therapeutic resistance (9, 20, 23). However, most existing studies focus on individual lncRNA–target interactions or isolated signaling pathways, while a systematic understanding of how distinct microenvironmental stresses converge through shared lncRNA-dependent regulatory circuits remains incomplete. In particular, the coordination between four major stress conditions—nutrient deprivation, oxidative/ferroptotic stress, immunosuppressive microenvironmental stress, and therapeutic pressure—and four major lncRNA regulatory mechanisms—ceRNA regulation, protein scaffolding, chromatin regulation, and stress sensing—requires further integration. Moreover, the functional hierarchy of lncRNAs within stress-response pathways—whether they act as upstream stress sensors or downstream effectors of adaptive programs—has not been systematically delineated.

On these grounds, this review moves beyond cataloging individual lncRNA–target interactions to construct a unifying framework that links four major stress conditions with four lncRNA regulatory mechanisms. We propose that lncRNAs function as stratified nodes within a cross-stress adaptation network, and we distinguish stress-responsive regulatory relays from downstream effectors while recognizing that these roles can overlap and switch depending on cell type, genetic background, stress intensity, and treatment context. This review also summarizes the translational implications of stress-related lncRNAs as biomarkers and therapeutic targets, while outlining current bottlenecks and future directions for lncRNA-based precision medicine in NSCLC. This review is organized as follows: Chapter 2 introduces the four canonical molecular mechanisms of lncRNA regulation; Chapter 3 systematically dissects lncRNA-mediated adaptation across four major stress categories and their cross-compensatory network; Chapter 4 evaluates clinical translational potential, including biomarkers, therapeutic targeting, combination strategies, and translational barriers; and Chapter 5 outlines current research gaps and future directions.

### 1.1 Search strategy and selection criteria

Relevant literature for this narrative review was identified through searches of PubMed, Embase, and Web of Science up to October 5, 2026. Disease-related terms, including “non-small cell lung cancer”, “NSCLC”,”lung adenocarcinoma”,and “lung squamous cell carcinoma” were combined with lncRNA-related terms, including “long non-coding RNA”, “lncRNA”, and “lincRNA”.Topic-specific terms included “hypoxia”, “nutrient deprivation”, “metabolic stress”,”oxidative stres”, “ferroptosis”, “lipid peroxidation”, “immune evasion”,”autophagy”, “DNA repair”, “drug resistance”, “EGFR-TKI resistance” and “radioresistance”. Boolean operators AND and OR were used to combine terms as appropriate. Studies published within the five years preceding the final search were prioritized, but publication year was not used as a strict exclusion criterion. Earlier publications were retained when they provided foundational findings or representative evidence relevant to the review.

Titles and abstracts were screened for relevance, followed by full-text assessment of potentially relevant articles. Peer-reviewed, English-language primary studies addressing lncRNAs in NSCLC in the context of metabolic, oxidative/ferroptotic, hypoxic/immune, or therapeutic stress were considered for inclusion. Review articles were used for background and contextual synthesis, whereas primary studies were prioritized to support specific mechanistic claims. Non-English publications, conference abstracts, and non-peer-reviewed preprints were excluded. Studies outside the central scope were considered only when they informed general mechanistic principles or methodological and translational limitations. Selected full-text articles were imported into Zotero for reference management and generation of the reference list.

The evidence was discussed with attention to lncRNA identity, histological subtype, driver alterations where reported, stress conditions, experimental models, proposed mechanisms, and the extent of validation. Bioinformatic studies without experimental validation were considered as association-based or hypothesis-generating evidence rather than confirmation of causal mechanisms or therapeutic efficacy. Clinical biomarker associations were distinguished from preclinical intervention findings. This review provides a narrative synthesis rather than an exhaustive systematic review, and the possibility of selection bias cannot be fully excluded.

## 2 Molecular mechanisms and stress-responsive roles of lncRNAs

According to their molecular interactions and functional characteristics, lncRNAs are mainly classified into four regulatory modes: competing endogenous RNA (ceRNA) regulation, protein scaffolding, chromatin epigenetic modulation, and stress-responsive regulation. These four mechanisms provide a unified framework for understanding how lncRNAs mediate tumor stress adaptation and malignant progression.

### 2.1 Proposed ceRNA regulation and its evidential requirements

The ceRNA mechanism represents one of the most extensively investigated regulatory modes of lncRNAs. Its fundamental principle is based on endogenous RNA competition, in which cytoplasmic lncRNAs containing microRNA response elements competitively bind free microRNAs, thereby reducing microRNA-mediated repression of downstream target mRNAs. Through this mechanism, lncRNAs indirectly enhance target gene expression by preventing microRNA binding to the 3′ untranslated region (3′UTR) of target transcripts and relieving translational inhibition or mRNA degradation (24, 25).

The regulatory efficiency of ceRNA networks depends on several molecular parameters, including the binding affinity between lncRNAs and microRNAs, intracellular abundance of competing molecules, and cellular localization patterns (26). A single lncRNA may interact with multiple microRNAs, whereas one microRNA may simultaneously be regulated by multiple lncRNAs, resulting in highly interconnected post-transcriptional regulatory networks (27). Building on this, ceRNA regulation provides tumor cells with a rapid and flexible mechanism for adapting gene expression programs under environmental stress.

In NSCLC, several studies have proposed ceRNA mechanisms involving metabolic remodeling, oxidative balance, hypoxic adaptation, and therapeutic resistance. For example, lncRNA ANRIL promotes glycolytic reprogramming through sequestration of miR-186-5p, thereby enhancing HIF-related signaling and tumor progression (28). PVT1 facilitates cisplatin resistance through interaction with miR-140-3p, bridging hypoxia-associated adaptation with chemotherapy tolerance (29). Furthermore, Uc.339 has been reported to regulate pri-miR-339 processing, illustrating a non-canonical miRNA-associated mechanism (25). These reports suggest possible miRNA-associated regulatory mechanisms, although their physiological interpretation depends on the validation available in each study.

The physiological relevance of proposed ceRNA interactions requires quantitative evaluation. Competition depends on the abundance of the lncRNA, the relevant AGO-loaded miRNA, the number and affinity of accessible binding sites, and the broader pool of competing targets (26, 139, 140). A large fold change in a low-abundance lncRNA may still provide insufficient binding capacity to alter miRNA-mediated repression. Subcellular colocalization is also necessary, and total-cell expression measurements may not represent effective concentrations within the relevant compartment.

Supraphysiological overexpression can create a competitive regime that is absent under endogenous conditions. A positive dual-luciferase assay supports sequence-dependent regulation in the reporter system but does not establish physiologically meaningful competition. Stronger evidence requires absolute transcript quantification, preferably expressed as copies per cell, localization analysis, and endogenous perturbation coupled to quantitative measurements of target mRNA and protein derepression. AGO-CLIP/HITS-CLIP or related approaches can support endogenous AGO-associated interactions, but binding evidence alone is insufficient to establish competition. Mutation of the relevant response elements and rescue at near-endogenous expression levels provide additional tests of specificity.

Accordingly, the miRNA-associated axes summarized here should be interpreted according to the evidence available in each primary study. Where endogenous stoichiometry and competition have not been demonstrated, these axes represent proposed mechanisms rather than established quantitative ceRNA circuits. This limitation concerns the proposed competitive mechanism and should not be interpreted as evidence that low-abundance lncRNAs necessarily lack biological functions.

### 2.2 Protein scaffold-mediated regulatory function

Beyond miRNA-mediated regulation, lncRNAs also act as molecular scaffolds by organizing protein complexes. Due to their modular RNA structures, individual lncRNA transcripts contain multiple protein-binding domains that allow simultaneous recruitment of different regulatory proteins. Through these interactions, lncRNAs regulate protein stability, enzymatic activity, signal complex assembly, and intracellular localization (30).

Protein scaffold functions mainly involve two regulatory patterns. First, lncRNAs interact with ubiquitination-related proteins to prevent degradation of key signaling molecules. For example, HOXC-AS3 binds the E3 ubiquitin ligase MDM2, thereby stabilizing YBX1 protein expression and enhancing oncogenic signaling activity (31). Second, lncRNAs serve as molecular bridges between signaling components, facilitating the assembly of functional protein complexes and amplifying downstream signaling cascades (32).

Unlike ceRNA regulation, which mainly occurs in the cytoplasm, scaffold-mediated regulation is not restricted to a specific cellular compartment. Nuclear and cytoplasmic lncRNAs both regulate protein complex organization depending on their molecular partners and biological context (30, 32). In NSCLC, MYLK-AS1, PURPL, and ScaRNA2 represent typical examples of scaffold-mediated regulation. These lncRNAs modulate glutamine metabolism, ferroptosis regulation, and DNA damage repair, respectively, thereby enhancing tumor cell survival under metabolic or therapeutic stress conditions (33–36).

### 2.3 Chromatin epigenetic modulation function

A major function of nuclear lncRNAs involves regulation of chromatin states and epigenetic programs. Through sequence-specific interactions with genomic regions, lncRNAs recruit chromatin-modifying complexes, including histone acetyltransferases, methyltransferases, and demethylases, to specific promoters or enhancer regions. These interactions develop transcriptional activation or repression programs at the chromatin level (37).

Compared with ceRNA and protein scaffold mechanisms, epigenetic regulation generally produces more sustained effects because chromatin modifications may persist after the initial environmental stimulus disappears. This property enables tumor cells to maintain long-term adaptive states and contributes to maintenance of cancer stemness and therapy resistance (38).

UCA1 provides a representative example of lncRNA-mediated chromatin regulation. Nuclear UCA1 interacts with EZH2 and recruits it to the CDKN1A promoter region, resulting in H3K27me3 deposition and transcriptional repression of cell-cycle inhibitory genes (39). In addition, LINC00969 demonstrates the potential role of epigenetic memory in drug resistance. Following gefitinib exposure, LINC00969-associated histone modifications persist after drug withdrawal, continuously suppressing pyroptosis-related pathways and maintaining a resistant phenotype (40).

Through stable remodeling of transcriptional programs, epigenetic lncRNA regulation influences metabolism, immune adaptation, and therapeutic resistance-associated gene expression, providing a molecular basis for persistent malignant phenotypes (41).

### 2.4 Stress-responsive lncRNAs as regulatory relays and effectors

Stress responsiveness describes the position of an lncRNA within a regulatory circuit rather than a molecular mechanism equivalent to miRNA competition, protein binding, or chromatin regulation. An increase in lncRNA expression after hypoxia or nutrient deprivation establishes stimulus responsiveness but does not, by itself, demonstrate direct sensing of oxygen, metabolites, or reactive oxygen species. We therefore distinguish stress-responsive regulatory relays from downstream effectors, while recognizing that these roles can overlap.

Several studies have provided direct evidence supporting stress-responsive characteristics of lncRNAs. Glucose deprivation induces AC020978 expression, which promotes metabolic adaptation under nutrient limitation. Hypoxic conditions regulate ANRIL expression, whereas cisplatin treatment alters PVT1 and GAS5 expression profiles, demonstrating that different environmental stimuli induce specific lncRNA responses (42–45).

Stress-responsive lncRNAs can connect environmental changes with downstream adaptive programs (38). However, stimulus-induced changes in lncRNA expression do not, by themselves, establish direct sensing of the initiating stress. In this review, stress responsiveness and downstream effector activity are therefore treated as overlapping functional dimensions rather than mutually exclusive categories. A given lncRNA may respond to an upstream stress signal and subsequently regulate adaptive processes through RNA–protein interactions, miRNA-associated regulation, or chromatin modulation. Its position within this regulatory circuit should be interpreted in relation to the stimulus, cellular context, and available experimental evidence.

Importantly, these four regulatory modes are not mutually exclusive. A single lncRNA may simultaneously function through ceRNA activity, protein scaffolding, epigenetic regulation, and stress-responsive regulation, forming multilayered regulatory networks that jointly determine tumor cell plasticity and survival under stress conditions (46). Drawing on this mechanistic framework, subsequent sections will discuss how lncRNAs regulate NSCLC adaptation to metabolic stress, oxidative/ferroptotic stress, microenvironmental stress, and therapeutic stress.

The representative lncRNAs executing these four regulatory modes under NSCLC microenvironmental stresses are summarized in **Table 1**.

**Table 1:** Representative lncRNAs with four core regulatory mechanisms in NSCLC.

Functions	lncRNA	Targets	Molecular mechanism	Microenvironmental stress	Reported biological effect	Evidence level
ceRNA sponges	ANRIL (28)	miR-186-5p,HIF-1α	Exosomal ANRIL was reported to sponge miR-186-5p, up-regulating HIF-1α	Oxidative stress & Drug induced stress	Promote cell proliferation, enhance aerobic glycolysis	*In vitro* + *In vivo*
	PVT1 (29)	miR-140-3p,ATG5	PVT1 was reported to sponge miR-140-3p, increasing ATG5 expression and activating autophagy	Oxidative stress & Drug induced stress	Induce autophagy, confer cisplatin chemoresistance and suppress cell apoptosis	*In vitro* + *In vivo*
	Uc.339 (25) (miRNA processing regulation)	miR-339,SLC7A11	Uc.339 competitively binds pri-miR-339 to cut down mature miR-339 and then upregulates SLC7A11 expression	Oxidative stress & Nutrient deprivation	Inhibit cell ferroptosis, promote cell proliferation, migration and invasion	*In vitro* + *In vivo*
	GAS5 (45)	miR-21,IGF-1R	GAS5 was reported to sequester miR-21 to repress IGF-1 expression	Drug induced stress	Inhibit proliferation, induce apoptosis and reverse gefitinib resistance	*In vitro*
Protein scaffolds	HOXC-AS3 (31)	YBX1,HOXC8	HOXC-AS3 binds YBX1 to block MDM2-mediated ubiquitination and degradation of YBX1, then YBX1 binds HOXC8 promoter to activate its transcription	No relevant stress types observed in this paper	Promote cell proliferation, migration and invasion	*In vitro*
	DUXAP9-206 (32)	Cbl-b,EGFR	DUXAP9–206 binds Cbl-b to block Cbl-b mediated EGFR ubiquitination and degradation then activates EGFR downstream signaling	No relevant stress types observed in this paper	Promote cell proliferation, migration and invasion	*In vitro*
	MYLK-AS1 (33)	ILF3,GLUD1	MYLK-AS1 drives ILF3 liquid-liquid phase separation to stabilize GLUD1 mRNA and boost glutamine metabolism	Nutrient deprivation & Drug induced stress	Promote cell proliferation, inhibit cell apoptosis, induce EGFR-TKI drug resistance	*In vitro* + *In vivo*
	PURPL (34)	RBM4,xCT	PURPL binds RBM4 to stabilize xCT mRNA and suppress macrophage ferroptosis	Oxidative stress & Immune suppressive microenvironment	Promote M2 macrophage polarization, inhibit ferroptosis	*In vitro* + *In vivo*
	ScaRNA2 (35)	ATR,Mre11,Exo1	ScaRNA2 scaffolds Mre11 and Exo1 to facilitate DNA end resection and activate ATR-mediated HR repair	Drug induced stress	Promote DNA damage repair, induce radiotherapy and chemoresistance	*In vitro*
	AC020978 (42)	PKM2	Stress-induced by HIF-1α, stabilizes PKM2 to boost glycolysis	Nutrient deprivation & Oxidative stress	Promote cell proliferation, migration and aerobic glycolysis	*In vitro* + *In vivo*
	SH3PXD2A-AS1 (47)	SIRT7, FOXM1	SH3PXD2A-AS1 binds SIRT7 and reduces succinylation of FOXM1 at K259, maintaining FOXM1 stability and activity	Drug induced stress	Promote cell proliferation, confer cisplatin resistance	*In vitro* + *In vivo*
Chromatin-guiding molecules	UCA1 (39)	EZH2,CDKN1A	UCA1 recruits EZH2 to CDKN1A promoter, mediates H3K27me3 histone modification and epigenetically represses CDKN1A transcription	Drug induced stress	Promote cell proliferation and migration, induce EGFR-TKI resistance, suppress cell cycle arrest and cell apoptosis	*In vitro* + *In vivo*
	HAS2-AS1 (86)	LSD1, EphB3	HAS2-AS1 recruits LSD1 to EphB3 promoter, alters H3K4me2 levels and suppresses EphB3 transcription	Drug induced stress	Promote cell proliferation, invasion, and gefitinib resistance	*In vitro* + *In vivo*
Chromatin-guiding molecules & Protein scaffolds	LINC00969 (40)	EZH2,METTL3,NLRP3	LINC00969 scaffolds EZH2/METTL3 to repress NLRP3 and inhibit pyroptosis	Drug induced stress	Promote cell proliferation and migration, inhibit pyroptosis, induce EGFR-TKI acquired resistance	*In vitro* + *In vivo*

NR, not reported or not established in the cited study. Stress responsiveness and effector mechanism are annotated as separate dimensions. Stress-induced changes in lncRNA abundance do not establish direct environmental sensing. A transcript may act as both a stress-responsive relay and an effector, depending on the experimental context. Genomic annotations identify the experimental context and do not imply that genotype dependence was demonstrated. Information obtained from external cell-line resources should be distinguished from genotyping performed in the primary study. Clinical expression or survival associations are not evidence of therapeutic efficacy. Evidence of a phenotype *in vivo* does not necessarily establish every proposed molecular interaction.

## 3 lncRNA-mediated multistress adaptation in NSCLC

Each lncRNA-associated phenotype should be interpreted within the histology, cell lineage, driver background, and treatment conditions of the underlying model. Findings in LUAD-derived models cannot be assumed to apply to LUSC, and observations in one resistant derivative do not establish a universal resistance mechanism. Where the primary report does not characterize relevant alterations, the genomic context should be regarded as unresolved rather than wild type.

The aberrant vascular architecture of non-small cell lung cancer (NSCLC), together with sustained tumor cell proliferation and anticancer treatment, creates a dynamically changing environment characterized by multiple concurrent stresses. Nutrient limitation, redox imbalance, hypoxia and acidosis, and immunosuppression frequently coexist and are interconnected through metabolites, reactive oxygen species (ROS), cytokines, and intercellular communication. Tumor cells adapt to hypoxia and nutrient deprivation by reprogramming glycolysis, oxidative phosphorylation, lipid metabolism, and amino acid metabolism, and this metabolic plasticity is closely associated with redox homeostasis and therapeutic tolerance (48). Moreover, adaptive programs established under individual stress conditions, including metabolic rewiring, antioxidant defense, autophagy, DNA repair, and immune evasion, may subsequently intersect and cooperate under therapeutic pressure. Consequently, metabolic, oxidative, microenvironmental, and treatment-related stresses do not operate in isolation; rather, they constitute an interconnected and compensatory adaptive network, wherein feedback and cross-compensation prevail over linear, single-pathway responses.

Within this network, lncRNAs operate at distinct regulatory levels. Some undergo changes in expression, chemical modification, or subcellular localization in response to defined stimuli—glucose deprivation, hypoxia, or therapeutic exposure—and thus function at a relatively upstream stress-response level. Others execute adaptive programs through competing endogenous RNA (ceRNA) activity, RNA–protein interactions, chromatin regulation, or epitranscriptomic mechanisms. These regulatory modes are not mutually exclusive, and a single lncRNA may engage multiple mechanisms simultaneously. Accordingly, this chapter is organized around four major categories of stress. It focuses on the continuous regulatory trajectory from “stress input” to “lncRNA response,” “molecular execution,” “adaptive phenotype,” and ultimately “cross-stress outcome”.

Hence, lncRNAs can be positioned along a functional continuum between stress input and adaptive output: those closer to the input end function as stress sensors or upstream relays, whereas those closer to the output end act as downstream effectors, and many lncRNAs operate at both levels simultaneously.

### 3.1 Metabolic stress adaptation: nutrient deprivation-driven metabolic reprogramming

Rapid proliferation and inadequate perfusion expose local regions of NSCLC tumors to persistent fluctuations in the availability of nutrients such as glucose and glutamine. To sustain ATP production, biosynthesis, and redox homeostasis, tumor cells redistribute metabolic flux across glycolysis, glutamine metabolism, and mitochondrial oxidative metabolism (48, 49). lncRNAs participate at different levels of this process: some directly respond to nutrient stress, whereas others primarily function as downstream effectors of metabolic reprogramming.

#### 3.1.1 Stress-sensing lncRNAs initiate nutrient-stress signaling

Declining energy and nutrient availability is initially detected by canonical sensing systems, including AMPK and metabolite-sensitive enzymes (49), in turn reshaping transcriptional regulation and RNA/chromatin modifications. Glucose deprivation provides a direct model linking metabolic stress to lncRNA alterations. Low-glucose conditions affect the activities of α-ketoglutarate (α-KG)-dependent enzymes and the RNA demethylase FTO, resulting in changes in the methylation and expression of a subset of lncRNAs. Among these, LINC00662 facilitates EZH2 recruitment to target promoters under glucose deprivation, coupling nutrient availability to H3K27me3-driven transcriptional programs (15); thereby serving as a molecular bridge transducing metabolic stress signals into durable epigenetic adaptations.

AC020978 also exhibits a well-defined nutrient-responsive profile. Low glucose and 2-deoxy-D-glucose (2-DG) induce AC020978 expression in a time- and dose-dependent manner, while hypoxia further increases its abundance. AC020978 directly binds to and stabilizes PKM2, promotes its nuclear translocation, and enhances PKM2/HIF-1α-associated transcriptional activity, ultimately increasing glycolytic flux (42). This pathway illustrates how glucose deprivation and hypoxia can converge on the AC020978/PKM2/HIF-1α axis to establish a shared node of metabolic adaptation (see **Table 1**).

By contrast, LINC01123 primarily amplifies an already activated glycolytic program through a positive-feedback circuit. c-Myc directly activates LINC01123 transcription, whereas LINC01123 competitively binds miR-199a-5p and relieves its repression of c-Myc. This LINC01123/miR-199a-5p/c-Myc feedback loop subsequently increases the expression of glycolysis-related factors such as HK2 and LDHA (50). Accordingly, LINC01123 is clearly involved in glycolytic reprogramming, with c-Myc representing its principal upstream driver identified to date.

In addition, cancer-associated fibroblast (CAF)-derived exosomal ANRIL promotes NSCLC glycolysis and proliferation by sponging miR-186-5p and thereby relieving repression of HIF-1α (28). This mechanism demonstrates how stromal cells can transmit metabolic regulatory signals to tumor cells through exosomal lncRNAs and reinforce HIF-1α-dependent glycolytic programs.

Collectively, lncRNAs involved in metabolic stress can be broadly separated into two functional levels: those whose expression or modification changes directly in response to nutrient stress and those that primarily amplify or execute pre-existing metabolic programs through ceRNA activity, protein interactions, or related mechanisms.

#### 3.1.2 Reported miRNA-associated mechanisms in metabolic adaptation

In metabolic adaptation, ceRNA regulation has been proposed as one mechanism through which lncRNAs influence the expression of metabolic enzymes, transcription factors, and mitochondrial transporters (48) By competing for miRNAs, these networks translate changes in lncRNA abundance into altered expression of metabolic enzymes, transcription factors, and mitochondrial transport proteins.

The LINC01123/miR-199a-5p/c-Myc axis is a representative positive-feedback circuit controlling glycolysis. By releasing c-Myc from miR-199a-5p-mediated repression, LINC01123 increases HK2 and LDHA expression and enhances aerobic glycolysis (50). PCIF1 similarly promotes glycolysis through the miR-326/PKM axis and contributes to cisplatin tolerance in A549/DDP cells (51). This pathway as such links glycolytic reprogramming directly to therapeutic resistance, illustrating how metabolic programs can be repurposed under treatment pressure.

Glutamine utilization is also regulated by ceRNA networks. FEZF1-AS1 competitively binds miR-32-5p, increases GLS expression, enhances glutaminolysis, and contributes to cisplatin resistance (52). These observations further indicate that metabolic reprogramming not only enables adaptation to nutrient fluctuations but also provides metabolic buffering during anticancer treatment.

Mitochondrial oxidative metabolism is regulated through the LINC02802 axis. NAT10-mediated N4-acetylcytidine (ac4C) modification stabilizes LINC02802, which subsequently sponges miR-1976 and upregulates the mitochondrial NAD^+^ transporter SLC25A51. This pathway maintains the mitochondrial NAD^+^/NADH balance and sustains oxidative tricarboxylic acid (TCA) cycle flux (53), thereby establishing a multilayered regulatory cascade comprising ac4C modification, lncRNA stabilization, ceRNA activity, and mitochondrial metabolism.

Lipid metabolism can likewise be influenced by extracellular-vesicle-mediated lncRNA–ceRNA networks. ROLLCSC, carried by extracellular vesicles released from lung cancer stem cells, regulates lipid metabolism through miR-5623-3p- and miR-217-5p-associated networks and enhances the plasticity and metastatic potential of non-stem tumor cells (54).

Overall, metabolism-associated ceRNA networks in NSCLC converge on c-Myc, PKM, GLS, SLC25A51, and lipid metabolic pathways, thereby coordinating glycolysis, glutamine utilization, mitochondrial oxidative metabolism, and lipid metabolism in parallel.

#### 3.1.3 RNA–protein interactions in metabolic regulation

Beyond ceRNA activity, lncRNAs directly interact with RNA-binding proteins or metabolic enzymes, introducing an additional layer of spatial and molecular organization into metabolic regulation. Mir100hg represents a prominent example of such multimodal regulation. Mir100hg derived from lung cancer stem cells can be packaged into exosomes with the involvement of HNRNPF and HNRNPA2B1 and subsequently transferred to recipient cells (55). Once internalized, Mir100hg exerts two complementary effects: it competitively binds miR-15a-5p and miR-31-5p, while also interacting directly with ALDOA and recruiting the deubiquitinase OTUD4. These mechanisms reduce ALDOA ubiquitination and degradation, ultimately stabilizing ALDOA and enhancing glycolysis and lactate production (55).

This example illustrates that ceRNA regulation and RNA–protein interactions are not isolated regulatory modules. Instead, a single lncRNA can employ distinct mechanisms that converge on the same metabolic node.

LCAT1 provides another mode of RNA–protein regulation. LCAT1 directly binds to and stabilizes the m6A reader IGF2BP2, which in turn stabilizes CDC6 mRNA in an m6A-dependent manner and promotes lung cancer cell proliferation and malignant progression (56). This lncRNA–RNA-binding protein–m6A-targeted RNA stabilization axis provides an additional mechanistic framework through which lncRNAs contribute to metabolism-associated adaptation.

#### 3.1.4 Chromatin and epigenetic regulation sustain metabolic adaptation

Metabolic state and epigenetic regulation are bidirectionally interconnected. Under low-glucose conditions, changes in metabolite availability and the activity of metabolite-sensitive enzymes influence RNA and histone modifications, whereas LINC00662 facilitates EZH2 recruitment to target promoters, thereby converting nutrient alterations into relatively stable transcriptional states (15).

Beyond α-KG-dependent demethylases and FTO, glucose deprivation may also influence lncRNA regulation through AMPK-mediated phosphorylation of transcription factors and chromatin regulators, although direct evidence that these upstream events modulate lncRNA promoter accessibility in NSCLC remains limited. Similarly, nutrient stress has been proposed to alter the subcellular localization of certain lncRNAs through changes in nuclear-cytoplasmic transport; whether this constitutes a general post-transcriptional mechanism of rapid metabolic adaptation, however, awaits further experimental characterization.

Conversely, metabolites themselves serve as substrates or cofactors for epigenetic reactions. Acetyl-CoA supplies acetyl groups for histone acetylation, α-KG participates in a broad range of dioxygenase reactions, and metabolites such as succinate and fumarate can modulate demethylation processes. Metabolic reprogramming can therefore feed back onto chromatin organization and epigenetic state (57).

Mir100hg further illustrates a continuous regulatory trajectory from lncRNA activity to metabolic enzymes, metabolites, and epigenetic modification. By stabilizing ALDOA, Mir100hg increases glycolysis and lactate production and enhances H3K14 lactylation (55). These findings demonstrate that lncRNA-driven metabolic reprogramming can propagate into the epigenetic layer, thereby linking metabolic state to chromatin regulation.

#### 3.1.5 Metabolic reprogramming connects subsequent stress-adaptation programs

The significance of metabolic reprogramming extends well beyond ATP generation (48). Increased glycolysis elevates lactate production and promotes local acidification; alterations in mitochondrial metabolism and the NAD^+^/NADH balance affect cellular ROS burden; and glutamine and lipid metabolism contribute to antioxidant defense, membrane lipid composition, and treatment response. At the same time, metabolites can further reshape epigenetic regulation (57).

Metabolic stress can therefore be viewed as an important entry point into the broader multistress network. Changes in lactate, ROS, reducing equivalents, and epigenetic modifications can subsequently influence the ferroptotic threshold, the hypoxic and immune microenvironment, and therapeutic sensitivity rather than terminating at the metabolic level.

For instance, Mir100hg-driven glycolytic reprogramming increases lactate production and subsequently enhances H3K14 lactylation (55), illustrating how metabolic stress propagates into the epigenetic layer; likewise, alterations in mitochondrial NAD^+^/NADH balance mediated by the LINC02802 axis (53) can directly influence cellular ROS burden and thereby shift the ferroptotic threshold. These observations position metabolic stress as a critical entry point into the broader multistress network rather than a self-contained adaptive program (**Figure 1**).

**Figure 1:**
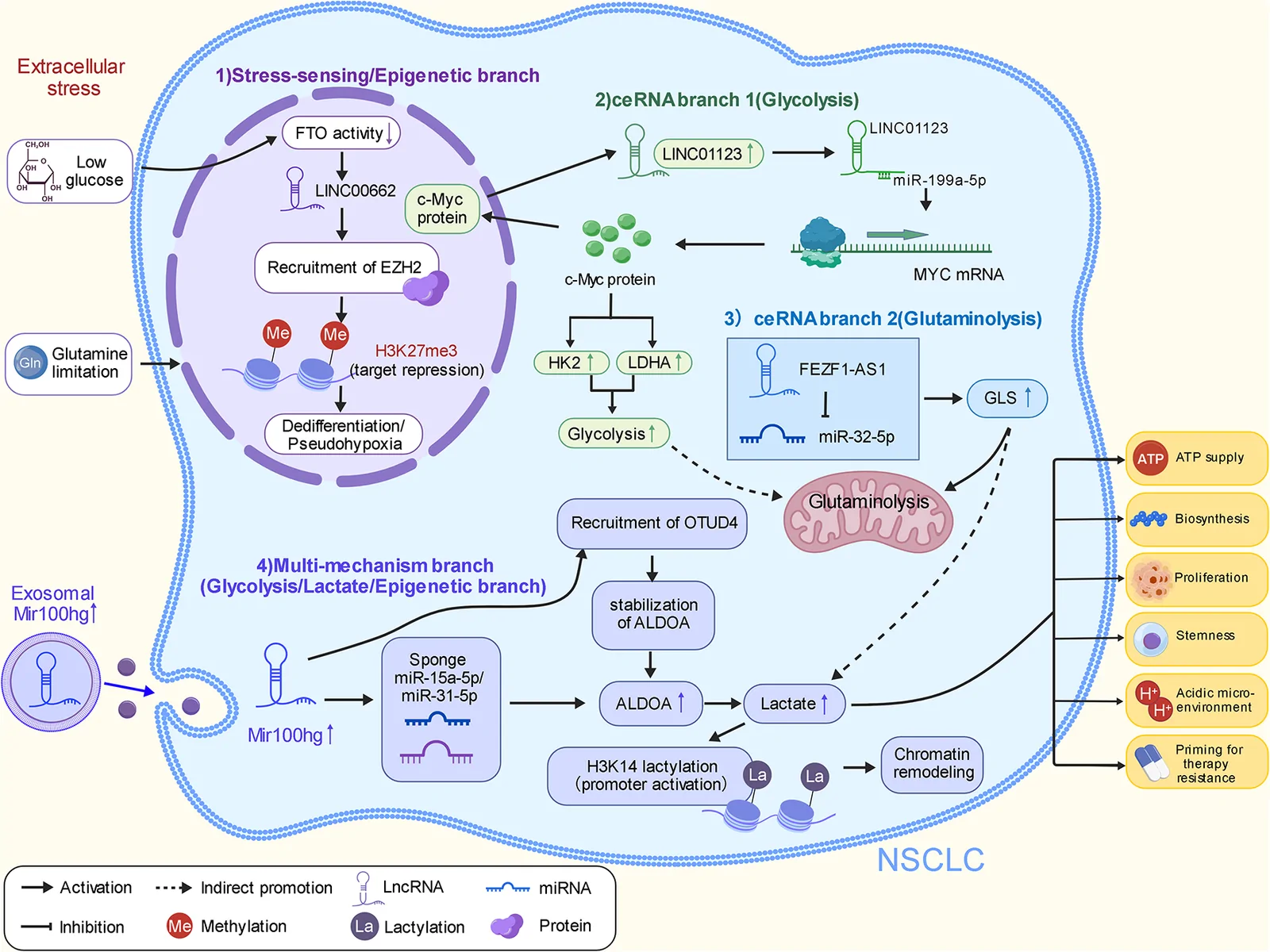
Metabolic stress adaptation in NSCLC. This image illustrates how lncRNAs coordinate metabolic adaptation in NSCLC under nutrient limitation and related metabolic stress. Nutrient limitation, particularly glucose and glutamine deprivation, initiates metabolic adaptation through stress-responsive, ceRNA, RNA–protein, and epigenetic regulatory mechanisms. Under low-glucose conditions, altered FTO activity and LINC00662-associated EZH2 recruitment link nutrient sensing to epigenetic remodeling. In parallel, the LINC01123/miR-199a-5p/c-Myc axis reinforces glycolytic signaling, whereas FEZF1-AS1 promotes glutamine utilization through the miR-32-5p/GLS pathway. Exosome-derived Mir100hg further integrates ceRNA regulation with RNA–protein interactions by promoting ALDOA stabilization, thereby increasing glycolysis and lactate production. Elevated lactate subsequently enhances H3K14 lactylation, linking metabolic reprogramming to chromatin remodeling. Together, these interconnected pathways sustain ATP production, biosynthesis, and metabolic plasticity, enabling NSCLC cells to adapt to nutrient stress and establishing a metabolic foundation for subsequent redox and therapeutic adaptation.

### 3.2 Oxidative stress adaptation: lipid peroxidation and ferroptosis regulation

A high metabolic burden, mitochondrial redox imbalance, chemotherapy, and radiotherapy can all increase cellular ROS levels. Ferroptosis is a regulated form of cell death driven by iron-dependent lipid peroxidation (58). The System Xc^−^/GSH/GPX4 axis constitutes a major anti-ferroptotic defense system, whereas SLC7A11, GPX4, ACSL4, iron homeostasis, and membrane lipid composition collectively determine cellular tolerance to lipid peroxidation (59–61).

Genomic background is particularly important when interpreting oxidative-stress and ferroptosis phenotypes. In KEAP1-inactive lung cancer models, NRF2-dependent induction of FSP1 provides a CoQ-associated ferroptosis defense pathway that can influence radiation response (141). Changes in SLC7A11 or GPX4 after lncRNA perturbation should therefore be interpreted alongside parallel antioxidant defenses and KEAP1–NRF2 pathway activity. STK11/LKB1 status should also be considered when evaluating metabolic and immune phenotypes. STK11 and KEAP1 alterations are distinct contextual variables and should not be treated as interchangeable (142).

For redox-associated lncRNAs, reporting the relevant genotype is an important first step. Demonstrating genotype dependence additionally requires comparisons across molecularly annotated models or appropriate isogenic perturbations. In their absence, conclusions should remain restricted to the tested experimental context rather than generalized across NSCLC.

#### 3.2.1 ROS responses and lncRNA-mediated regulation of the ferroptotic threshold

ROS accumulation activates canonical antioxidant responses, including the KEAP1–NRF2 pathway (62), whereas lncRNAs primarily modulate downstream antioxidant defenses, lipid peroxidation, and iron homeostasis to alter ferroptosis sensitivity.

SLC7A11AR was identified as a human-specific, inflammation-responsive lncRNA with preferential expression in the LUAD context, whereas comparable upregulation was not observed in the tested LUSC cell lines (16). The study linked SLC7A11AR to SLC7A11 expression through a reported miR-150-5p-associated mechanism. SLC7A11AR depletion increased lipid peroxidation, ROS, MDA, and Fe²^+^ while reducing GSH, consistent with increased ferroptosis susceptibility (16). These findings support a context-dependent regulatory mechanism rather than a conclusion applicable to all NSCLC subtypes.

MT1DP acts in the opposite direction. Rather than functioning through a canonical ceRNA mechanism, MT1DP binds to and stabilizes miR-365a-3p, thereby strengthening miR-365a-3p-mediated repression of NRF2. This increases ROS, MDA, and iron accumulation and enhances erastin-induced ferroptosis (63).

Bioinformatic and expression-correlation analyses further suggest that the GSEC/miR-101-3p/CISD1 axis may constitute a ferroptosis-associated regulatory network in LUAD, providing a candidate pathway for subsequent mechanistic investigation (64); however, this predicted axis remains to be experimentally validated.

#### 3.2.2 Reported miRNA-associated regulation of ferroptosis sensitivity

Ferroptosis-associated ceRNA networks have been reported to converge on SLC7A11, GPX4, and lipid metabolic pathways (59) thereby shaping the ferroptotic threshold through coordinated regulation of antioxidant capacity and lipid peroxidation.

Uc.339 was reported to bind pri-miR-339 and reduce the production of mature miR-339, thereby increasing SLC7A11 expression (25). This mechanism concerns regulation of miRNA processing and should be distinguished from canonical ceRNA competition for mature miRNA. Separately, SLC7A11AR has been linked to SLC7A11 expression and ferroptosis suppression through a reported miR-150-5p-associated mechanism (16).

HCP5 influences Fe²^+^, MDA, SLC7A11, ACSL4, and other ferroptosis-associated phenotypes in A549 cells through the miR-17-5p/HOXA7 axis (65), suggesting that this pathway participates in the coordinated regulation of ferroptotic status and malignant phenotypes in LUAD.

NEAT1 competitively binds miR-101-3p, increases CALCRL expression, and alters ROS levels, ATP production, mitochondrial membrane potential, and apoptosis (66). This pathway thereby links redox regulation to mitochondrial function and broader cell-survival programs.

#### 3.2.3 RNA–protein interactions and epigenetic regulation beyond ceRNA-mediated ferroptosis control

lncRNAs regulate ferroptosis not only through ceRNA activity but also through RNA–protein interactions, RNA modification, and transcriptional regulation.

LINC00336 combines RNA–protein interactions with ceRNA activity. It directly binds ELAVL1, which enhances LINC00336 stability, while simultaneously acting through the MIR6852/CBS axis to reduce intracellular iron, Fe²^+^, and lipid ROS, thereby suppressing ferroptosis (67). LINC00336 for this reason represents a typical example of coordinated multimodal regulation.

RGMB-AS1 primarily functions through protein interactions. It binds HMOX1 and prevents TRC8-mediated ubiquitination and degradation of HMOX1, while also interacting with NAA10 and enhancing its activity. Together, these mechanisms promote ferroptosis and suppress NSCLC growth (68).

RBMS3-AS3 further connects RNA modification, RNA-binding proteins, and GPX4 transcription. ALKBH5 reduces RBMS3-AS3 levels through an m6A-associated mechanism; RBMS3-AS3 subsequently binds HNRNPDL and promotes ZEB1 mRNA stability, while ZEB1 represses GPX4 transcription, ultimately increasing ferroptosis sensitivity (69).

MNX1-AS1 provides another example of crosstalk between ferroptosis and therapeutic response. MNX1-AS1 depletion increases ACSL4 expression and enhances RSL3-induced ROS generation, MDA accumulation, and ferroptosis, while simultaneously reducing ABCG2 expression and potentiating paclitaxel-induced apoptosis (70). This establishes a mechanistic link between oxidative stress and treatment-related stress. In sum, these findings demonstrate that RNA–protein interactions, RNA modification, and transcriptional regulation can modulate the ferroptotic threshold independently of canonical ceRNA mechanisms.

#### 3.2.4 Crosstalk between ferroptosis regulation and the immune microenvironment

Ferroptosis not only determines tumor-cell survival but also influences macrophage recognition, effector T-cell recruitment, and tumor immunogenicity through oxidized lipid mediators and damage-associated molecular patterns (DAMPs) (6). Conversely, the immune microenvironment can modulate ferroptosis sensitivity in tumor cells. For example, IFN-γ derived from CD8^+^ T cells represses SLC3A2/SLC7A11 expression and thereby enhances tumor-cell ferroptosis (6). Ferroptosis on this basis represents an important interface between oxidative stress and the immune microenvironment, and lncRNA-mediated regulation of the ferroptotic threshold may further contribute to bidirectional adaptation between tumor cells and their immune milieu.

PURPL provides an example of a reported connection between ferroptosis regulation and macrophage polarization. The PURPL/RBM4/xCT axis has been linked to stabilization of xCT mRNA, suppression of ferroptosis, and promotion of M2 macrophage polarization (34). In particular, xCT knockdown was reported to counteract the polarization phenotype through induction of macrophage ferroptosis. This evidence should be distinguished from a demonstrated tumor-cell-autonomous anti-ferroptotic mechanism. Its therapeutic interpretation therefore requires attention to the cellular compartment in which PURPL is perturbed and the resulting effects on tumor–immune interactions (**Figure 2**).

**Figure 2:**
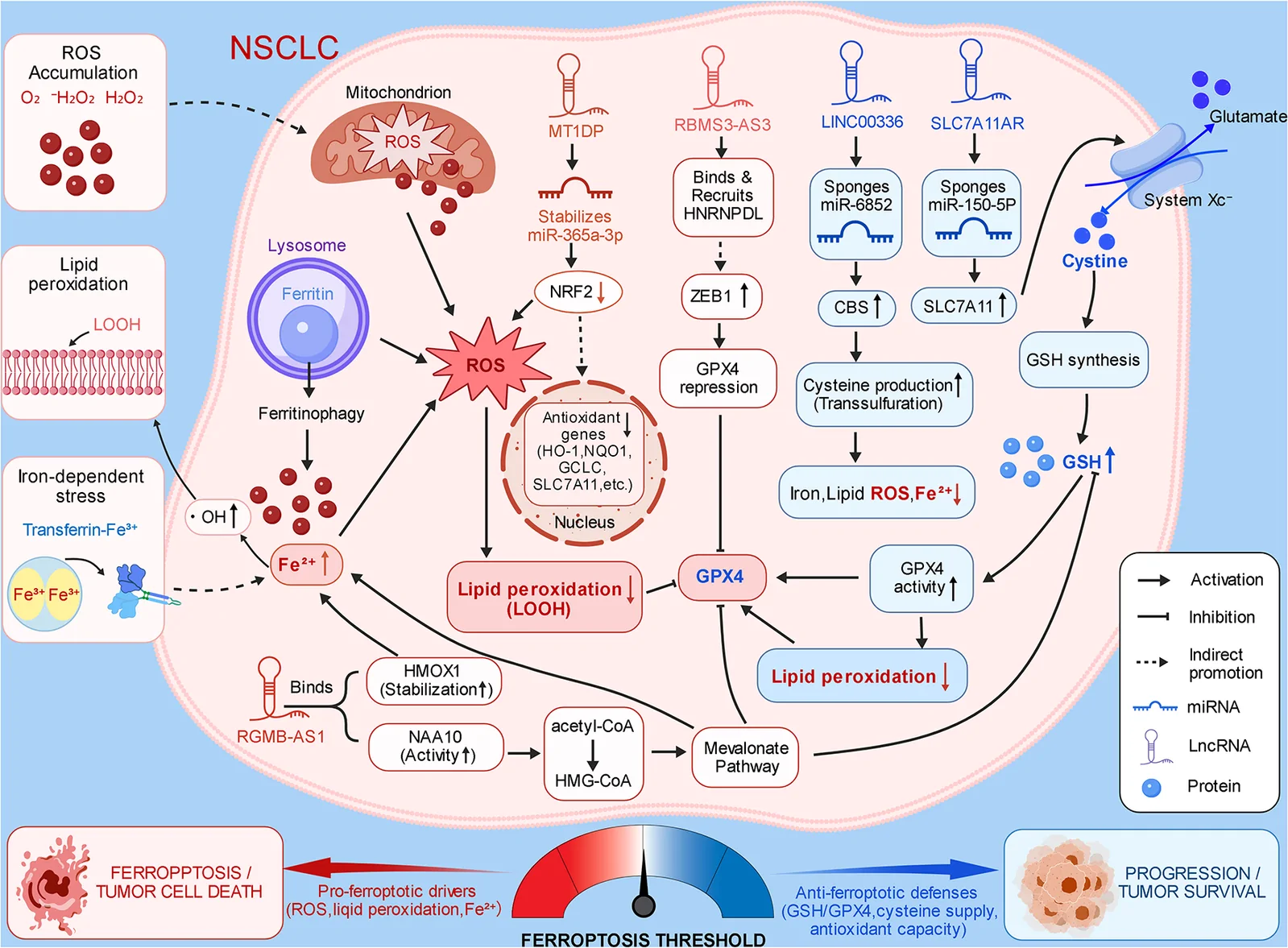
Oxidative stress adaptation and ferroptosis regulation in NSCLC. A schematic overview of lncRNA-mediated regulation of oxidative stress adaptation and the ferroptotic threshold in NSCLC. ROS accumulation, mitochondrial dysfunction, iron-dependent stress, and lipid peroxidation constitute major upstream pressures that determine ferroptotic susceptibility. lncRNAs regulate this process through multiple convergent mechanisms. SLC7A11AR enhances SLC7A11-dependent System Xc^−^ activity, promoting cystine uptake, glutathione synthesis, and GPX4-mediated detoxification of lipid peroxides, whereas LINC00336 reinforces anti-ferroptotic signaling through ceRNA and RNA–protein interactions. RBMS3-AS3 regulates the HNRNPDL/ZEB1/GPX4 axis, further influencing antioxidant capacity. In contrast, MT1DP suppresses NRF2-dependent antioxidant defense and thereby increases oxidative stress and ferroptotic susceptibility. RGMB-AS1 additionally connects ferroptosis regulation with HMOX1 stabilization and NAA10-associated metabolic signaling. The balance among ROS generation, iron availability, antioxidant defense, and lipid peroxidation ultimately determines whether NSCLC cells undergo ferroptotic death or acquire enhanced resistance to oxidative stress.

### 3.3 Microenvironmental stress adaptation: hypoxia, acidic TME, and immune evasion

Aberrant vascular architecture causes persistent hypoxia, whereas enhanced glycolysis and lactate export further promote the development of an acidic tumor microenvironment (TME). Hypoxia, metabolic products, and immune-cell remodeling collectively establish an immunosuppressive niche (71). lncRNAs orchestrate this process through HIF signaling, ceRNA activity, immune-checkpoint regulation, and extracellular-vesicle-mediated intercellular communication (72).

#### 3.3.1 Hypoxia-responsive lncRNAs mediate adaptation to low oxygen

HIFs constitute one of the major transcriptional regulatory systems governing hypoxic responses. Multiple ncRNAs exhibit altered expression in hypoxic lung cancer environments (73). Among them, CASC15 displays a clear hypoxia-responsive profile. Exposure to 1% O_2_ induces a time-dependent increase in CASC15 expression, whereas HIF-1α depletion markedly attenuates this response. HRE mutagenesis and chromatin immunoprecipitation confirm that HIF-1α directly binds the CASC15 promoter to initiate its transcription (74). CASC15 in turn drives cis-expression of SOX4, which stabilizes β-catenin and potentiates Wnt-driven proliferation and migration (74).An independent NSCLC study also showed that CASC15 is highly expressed in tumor tissues and cells, with elevated expression associated with metastasis and poor prognosis. CASC15 depletion suppressed NSCLC cell proliferation, migration, and invasion and attenuated epithelial–mesenchymal transition (EMT)-associated phenotypes (75). Together, these findings establish CASC15 as a hypoxia-responsive lncRNA intimately linked to malignant NSCLC progression.

In addition to HIF-mediated transcriptional activation, hypoxia can alter lncRNA stability through hypoxia-induced changes in RNA-binding protein activity and microRNA abundance. Hypoxia also triggers global shifts in histone methylation and acetylation at lncRNA loci, providing an epigenetic layer of regulation that can sustain lncRNA expression even after oxygen levels are restored.

LINC00301 further connects hypoxic adaptation with remodeling of the immune microenvironment. FOXC1 promotes LINC00301 expression, after which LINC00301 enhances HIF-1α signaling through distinct cytoplasmic and nuclear mechanisms. These changes are accompanied by increased regulatory T-cell (Treg) abundance and reduced CD8^+^ T-cell infiltration, consistent with an immunosuppressive microenvironment (17). LINC00301 by extension represents a multilayer regulatory node linking amplification of hypoxic signaling to immune-microenvironment remodeling.

#### 3.3.2 Reported miRNA-associated regulation of HIF signaling and immune evasion

ceRNA networks operating under microenvironmental stress primarily converge on two classes of regulatory nodes: those that enhance HIF signaling and downstream adaptive programs, and those that directly affect immune checkpoints or immune-cell function.

At the level of HIF amplification, cytoplasmic LINC00301 competitively binds miR-1276 and relieves repression of HIF-1α, thereby strengthening HIF-1α-dependent hypoxic adaptation (17). This mechanism illustrates how ceRNA competition can directly transmit lncRNA alterations into the central HIF transcriptional network. SOX2-OT, in turn, competitively binds miR-30d-5p, increases PDK1 expression, activates mTOR, and promotes PD-L1 expression, thereby affecting CD8^+^ T-cell-associated antitumor immunity (76).

The MIR17HG/miR-17-5p/RUNX3 axis is likewise associated with immune evasion in NSCLC. MIR17HG directly regulates miR-17-5p, accompanied by changes in RUNX3 expression and Treg-related phenotypes (77), thereby linking lncRNA–miRNA regulation to immune-cell states.

Extracellular vesicles extend lncRNA regulation from the intracellular to the intercellular level. Exosomal lncRNAs derived from tumor cells, CAFs, and immune cells can be transferred among distinct cell populations and influence tumor metabolism, invasion, immune regulation, and therapeutic response (78). By this means, lncRNA regulation within the TME transcends intracellular ceRNA networks, encompassing intercellular signaling that bridges tumor, stromal, and immune compartments.

#### 3.3.3 RNA–protein and epigenetic regulation sustain microenvironmental adaptation

In addition to its cytoplasmic ceRNA activity, LINC00301 also operates in the nucleus, where it represses EAF2 through an EZH2-associated mechanism and modulates pVHL/HIF-1α regulation, resulting in increased HIF-1α accumulation (17). In this way, LINC00301 extends beyond post-transcriptional regulation to nuclear protein and epigenetic control, acting in concert with its cytoplasmic ceRNA activity to sustain HIF signaling.

Metabolic products can also transmit metabolic stress into the epigenetic layer. By stabilizing ALDOA, Mir100hg increases lactate production and subsequently enhances H3K14 lactylation (55). This establishes a continuous regulatory cascade linking lncRNA activity, metabolic enzymes, lactate production, and histone lactylation, thereby allowing metabolic alterations to propagate into epigenetic regulation. Viewed together, RNA–protein interactions, chromatin modifications, and metabolite-driven epigenetic changes constitute a non-ceRNA regulatory layer of hypoxic adaptation that cooperates with cytoplasmic ceRNA mechanisms to amplify microenvironmental adaptation.

#### 3.3.4 Cooperative amplification of immunosuppression by multiple microenvironmental factors

Lactate accumulation, persistent hypoxia, altered redox status, and immune-checkpoint activity do not occur independently. Lactate enhances HIF-1α-associated signaling and increase HIF-1α binding to the LAMC2 promoter, thereby promoting LAMC2 expression and malignant phenotypes (79). This illustrates how metabolic products can feed back to reinforce hypoxia-associated transcriptional programs.

Accordingly, metabolism, hypoxia, redox status, extracellular-vesicle-mediated communication, and immune-cell remodeling collectively shape the immunosuppressive TME (71, 72, 78) and may establish a microenvironmental basis that facilitates subsequent adaptation to therapeutic stress.

Importantly, this immunosuppressive and metabolically reprogrammed microenvironment also provides a preadaptive basis for subsequent therapeutic resistance: hypoxia-induced HIF signaling, lactate-driven acidification, and extracellular-vesicle-mediated intercellular communication can all be repurposed under chemotherapy, targeted therapy, or immune-checkpoint blockade, as discussed in the next section (**Figure 3**).

**Figure 3:**
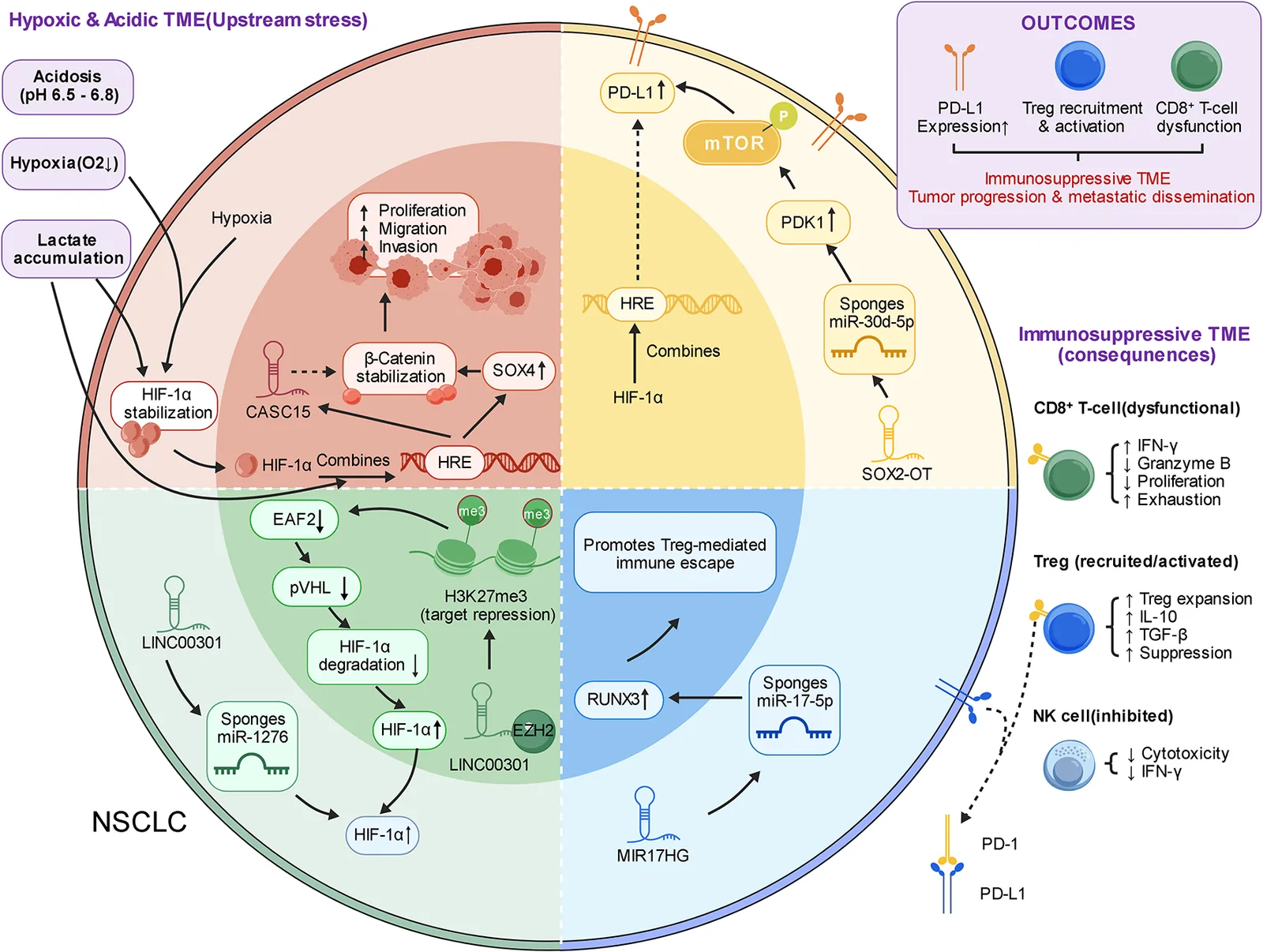
Hypoxic and acidic TME adaptation in NSCLC. Hypoxic and acidic microenvironmental signals are integrated through lncRNA-centered networks that couple tumor-cell adaptation with immune remodeling in NSCLC. Hypoxia, acidosis, and lactate accumulation act as interconnected upstream stresses that stabilize HIF-1α and reshape tumor–immune interactions. Hypoxia-induced HIF-1α activates CASC15, which promotes SOX4/β-catenin signaling and enhances tumor-cell proliferation and migration. LINC00301 further amplifies HIF-1α signaling through coordinated cytoplasmic ceRNA and nuclear regulatory mechanisms, thereby linking hypoxic adaptation to malignant progression. In parallel, immune-regulatory lncRNA networks extend these stress responses into the immune compartment. SOX2-OT activates the PDK1/mTOR pathway through miR-30d-5p sequestration, increasing PD-L1 expression, whereas the MIR17HG/miR-17-5p/RUNX3 axis contributes to Treg-associated immune escape. These interconnected pathways promote PD-L1 expression, Treg-mediated immunosuppression, and impaired CD8^+^ T-cell activity, thereby establishing a microenvironment that supports NSCLC progression, immune evasion, and subsequent therapeutic adaptation.

### 3.4 Therapeutic stress adaptation: resistance to chemoradiotherapy and targeted therapy

Therapeutic stress is highly interconnected with the three preceding categories of stress. Adaptive programs involving metabolic reprogramming, antioxidant defense, autophagy, DNA repair, EMT, and immune evasion may all be repurposed following treatment exposure (80, 81). In this way, therapeutic resistance can arise from adaptive remodeling under sustained treatment selection, while also being influenced by pre-existing microenvironmental pressures and oncogenic signaling. lncRNAs govern these processes through ceRNA networks, RNA–protein interactions, chromatin regulation, changes in subcellular localization, and micropeptide coding (82).

#### 3.4.1 Upstream induction of therapy-associated lncRNAs

The upstream origins of therapy-associated lncRNAs are heterogeneous and can be broadly grouped into microenvironmental preadaptation, sustained treatment selection, and oncogenic signaling. These distinct inputs ultimately converge on downstream resistance programs involving autophagy, DNA repair, and epigenetic regulation.

Hypoxia can provide a microenvironmental basis for chemotherapeutic preadaptation. For example, HIF-1α directly promotes PVT1 transcription, linking hypoxic stress to subsequent autophagy-mediated cisplatin tolerance (29) (detailed in Section 3.4.2).

Sustained drug exposure also drives changes in lncRNA expression and subcellular localization. H19 is markedly upregulated in A549/DDP cells and becomes enriched in mitochondria; its depletion suppresses mitophagy and restores cisplatin sensitivity (83). UCA1 and LINC00969, meanwhile, participate in gefitinib resistance through epigenetic and post-transcriptional regulatory mechanisms, respectively (39, 40).

Oncogenic signaling represents another source of resistance-associated lncRNA induction. EGFR activation induces TRIDENT expression. TRIDENT binds TRIM28 and promotes its phosphorylation, thereby increasing chromatin accessibility and DNA-damage repair capacity and ultimately enhancing therapeutic tolerance (19). Via this route, diverse upstream stimuli can converge on distinct lncRNAs that feed into common resistance-execution programs.

#### 3.4.2 Reported miRNA-associated mechanisms of therapeutic resistance

In therapeutic resistance, ceRNA networks function as major effector axes that converge on four common downstream programs: autophagy, metabolic reprogramming, cell-survival signaling, and drug-response regulation.

Hypoxia-induced PVT1 competitively binds miR-140-3p, upregulates ATG5, and enhances autophagy, thereby increasing cisplatin tolerance (29). This pathway directly links hypoxic stress, ceRNA regulation, and autophagy-mediated chemoresistance (see **Table 1**).

GLIDR competitively binds miR-342-5p, increases PPARGC1A expression, and contributes to cisplatin resistance (84). Reducing PPARGC1A expression increases cisplatin sensitivity in resistant cells, whereas PPARGC1A elevation alone is insufficient to confer a complete resistant phenotype in otherwise sensitive cells (84). These findings indicate that ceRNA targets generally function as important components of a broader resistance network rather than as isolated determinants.

In osimertinib-resistant derivatives of EGFR exon 19 deletion-positive PC9 and HCC827 cells, LINC01278 depletion increased osimertinib sensitivity, with miR-324-3p/ZFX proposed as a contributing regulatory axis (85). LINC01278 depletion also inhibited proliferation and promoted apoptosis, whereas ZFX restoration counteracted these effects. These findings provide *in vitro* evidence in a selected EGFR-mutant acquired-resistance setting and should not be generalized to EGFR-wild-type NSCLC or all mechanisms of osimertinib resistance.

#### 3.4.3 RNA–protein interactions, epigenetic regulation, and organelle remodeling broaden the mechanistic basis of therapeutic resistance

Beyond ceRNA activity, lncRNAs can promote therapeutic resistance through RNA–protein interactions, epigenetic regulation, and subcellular functional remodeling. These mechanisms bypass miRNA competition and directly alter protein activity, transcriptional state, or organelle function, thereby increasing tumor-cell adaptability under sustained treatment pressure.

First, RNA–protein interactions can directly modify the stability or post-translational state of key proteins. SH3PXD2A-AS1 (see **Table 1**) binds SIRT7 and reduces succinylation of FOXM1 at K259, thereby maintaining FOXM1 protein abundance and promoting cisplatin tolerance (47). This mechanism enables lncRNAs to act directly on resistance-effector proteins independently of ceRNA networks.

Second, lncRNAs can sustain resistance-associated gene-expression states through chromatin and epitranscriptomic regulation. HAS2-AS1 recruits LSD1 and alters H3K4me2 levels at the EphB3 promoter, suppressing EphB3 expression and promoting gefitinib resistance (86). LINC00969 further illustrates multilayered epigenetic regulation: on one hand, it increases H3K27me3 at the NLRP3 promoter through EZH2 and represses NLRP3 transcription; on the other, it regulates the post-transcriptional fate of NLRP3 through METTL3-mediated m6A modification. Together, these mechanisms reduce NLRP3 pathway activity and enhance gefitinib resistance (40). Epigenetic regulation can effectively operate at both transcriptional and post-transcriptional levels to prolong and diversify resistance programs.

Finally, lncRNAs can reshape organelle function through altered subcellular localization or even micropeptide translation. In cisplatin-resistant cells, H19 redistributes from the nucleus to mitochondria and supports mitophagy, thereby enhancing cell survival; H19 depletion restores cisplatin sensitivity (83). By contrast, AFAP1-AS1 encodes ATMLP, a mitochondria-localized micropeptide that dampens irradiation-triggered ROS, sustains AKT prosurvival signaling, and drives lipid-droplet biogenesis, rendering NSCLC cells resistant to radiotherapy (87).

#### 3.4.4 Multistress compensation shapes therapeutic tolerance

Therapeutic resistance rarely results from a single lncRNA or a single molecular pathway; rather, it emerges from the combined activity of multiple stress-adaptation programs. Hypoxia-induced PVT1 increases cisplatin tolerance through autophagy (29), and MYLK-AS1 promotes EGFR-TKI resistance by enhancing glutamine metabolism (33). MNX1-AS1 connects ferroptosis sensitivity with paclitaxel response (70), while ATMLP reduces radiotherapy-induced ROS and promotes AKT-driven lipid-droplet metabolism to increase radioresistance (87). Together, these mechanisms show that hypoxic, metabolic, redox, and cell-death programs can form mutually compensatory networks under therapeutic pressure.

Beyond directly established resistance pathways, lncRNAs can also modulate malignant phenotypes associated with tumor-cell plasticity, thereby providing a favorable background for therapeutic selection. LOXL1-AS1 promotes NSCLC cell proliferation, migration, and invasion through the miR-122-5p/E2F3 axis (88), whereas OTX2-AS1 activates PI3K/AKT/Snail signaling and promotes EMT through the miR-377-5p/EIF5A2 axis (89). Proliferation, migration, and EMT are closely associated with cell-state remodeling during resistance evolution and may increase the adaptive and selective capacity of tumor-cell populations.

Accordingly, therapeutic adaptation in NSCLC is better conceptualized as a dynamic network comprising metabolic reprogramming, redox regulation, hypoxic responses, DNA repair, autophagy, and cell-death programs (80–82, 90). Different lncRNAs act at distinct nodes within this network but ultimately converge on sustained tumor-cell survival and therapeutic tolerance.

Therapeutic stress thus represents the convergence point of the three preceding stress categories. Metabolic rewiring, antioxidant defense, and immunosuppressive programs established under nutrient deprivation, oxidative stress, and hypoxia are all repurposed under treatment pressure. This reinforces the view that these four stress dimensions form an integrated compensatory network rather than isolated pathways (**Figure 4**).

**Figure 4:**
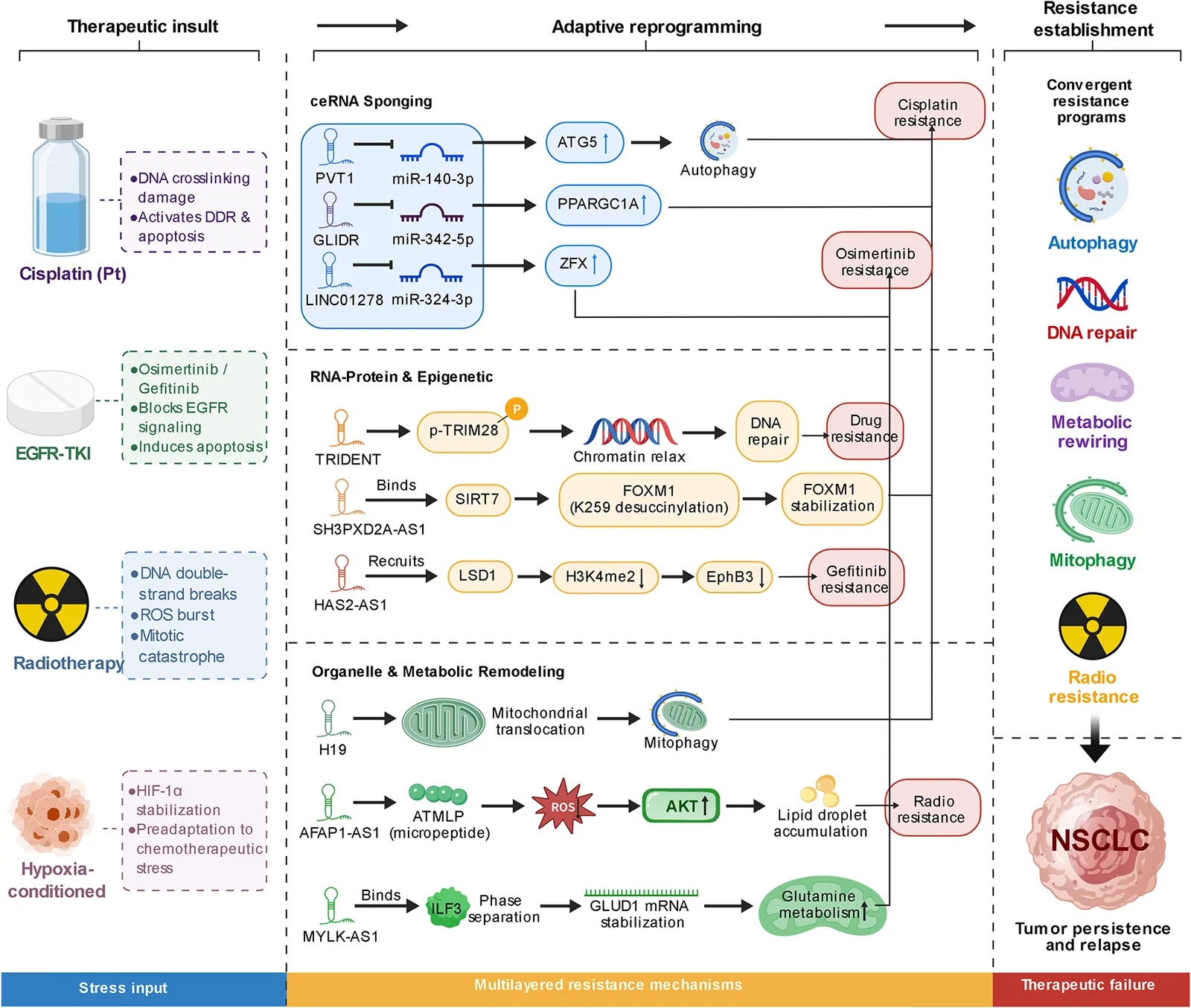
Therapy stress adaptation and resistance in NSCLC. Multiple therapeutic pressures converge on distinct but interconnected lncRNA-mediated resistance programs in NSCLC. Chemotherapy, EGFR-targeted therapy, radiotherapy, and pre-existing hypoxic stress impose distinct therapeutic pressures that converge on several lncRNA-dependent adaptive programs. At the ceRNA level, PVT1, GLIDR, and LINC01278 regulate ATG5, PPARGC1A, and ZFX, respectively, thereby promoting autophagy and resistance to cisplatin or osimertinib. RNA–protein and epigenetic mechanisms further reinforce resistance: TRIDENT promotes TRIM28-dependent chromatin remodeling and DNA-damage repair, SH3PXD2A-AS1 maintains FOXM1 stability through SIRT7-dependent regulation, and HAS2-AS1 recruits LSD1 to remodel the EphB3-associated transcriptional state. At the organelle and metabolic levels, H19 promotes mitochondrial mitophagy, AFAP1-AS1-derived ATMLP activates AKT signaling and lipid-droplet accumulation, and MYLK-AS1 enhances glutamine metabolism. These mechanisms ultimately converge on autophagy, DNA repair, metabolic rewiring, mitophagy, and prosurvival signaling, collectively driving persistent therapeutic resistance and NSCLC relapse.

### 3.5 Chapter summary

A central theme of this chapter is that NSCLC adaptation is governed by dynamic cross-compensation among stress programs rather than persistent activation of any single pathway. Nutrient deprivation and hypoxia initiate metabolic rewiring (48), which subsequently alters redox balance and epigenetic states (57). These adaptive programs are then repurposed during chemotherapy, targeted therapy, and radiotherapy (80, 82).

Within this network, lncRNAs exhibit clear functional stratification. Some directly execute adaptive programs through ceRNA competition, RNA–protein interactions, chromatin regulation, or RNA modification, whereas others influence organelle and intercellular states through altered subcellular localization, extracellular-vesicle-mediated communication, or micropeptide coding. lncRNAs also occupy different positions within the stress-response hierarchy. Some exhibit a continuous regulatory chain from environmental stimulus to adaptive phenotype, exemplified by low-glucose-associated LINC00662, glucose/hypoxia-responsive AC020978, and HIF-1α-regulated CASC15 (15, 42, 74). Many others act primarily as downstream effectors that amplify or maintain metabolic adaptation, ferroptosis regulation, immune modulation, and therapeutic resistance. This functional stratification positions lncRNAs as regulatory nodes at distinct levels between “stress input” and “adaptive output,” and provides a mechanistic rationale for combinatorial strategies designed to dismantle multiple adaptive programs simultaneously—an idea further developed in the following chapter on clinical translation.

## 4 Clinical translational potential of stress-related lncRNAs

The preceding chapters have shown that lncRNAs undergo changes in expression or function in response to hypoxia, nutrient deprivation, oxidative stress, and therapeutic exposure, while also participating in adaptive programs such as metabolic reprogramming, autophagy, DNA repair, cell death, and immune evasion (91, 92). Stress-related lncRNAs combine the capacity to reflect tumor states with direct regulatory functions, conferring potential translational value for diagnosis, prognostic assessment, and therapeutic intervention.

### 4.1 Diagnostic and prognostic biomarkers

lncRNAs exhibit pronounced tissue-, cell type-, and state-specific expression patterns, and their dysregulation is closely associated with NSCLC initiation, progression, and treatment response (91). Compared with single-molecule biomarkers, multi-lncRNA signatures integrate information from distinct biological processes and have increasingly been explored for risk stratification and prognostic assessment. For example, a stress-related lncRNA-based risk model stratifies patients with LUAD into prognostically distinct subgroups (93). In addition, lncRNAs interact extensively with major oncogenic signaling pathways, including Wnt/β-catenin and Hippo/YAP (94, 95). Integrating lncRNA expression profiles with pathway activity improves the biological interpretability of molecular classification.

Liquid biopsy further broadens the potential clinical applications of lncRNAs. Exosomes and other extracellular vesicles (EVs) transport lncRNAs between cells, while membrane encapsulation contributes to the stability of their RNA cargo. Lung cancer-derived exosomal lncRNAs have been associated with invasion and metastasis, angiogenesis, therapeutic resistance, and immune regulation (96). Hypoxia also alters exosome release and molecular cargo composition (97), suggesting that circulating RNAs capture, at least in part, changes in the tumor microenvironment. Studies in lung cancer further support the potential of circulating lncRNAs as noninvasive biomarkers (98).

In NSCLC, a plasma EpCAM-specific exosomal five-lncRNA panel comprising linc01125, HNF1A-AS1, MIR100HG, linc01160, and ZNRF3-AS1 has shown potential for early detection (99). In a separate cfRNA-Seq study, circulating mRNAs, lncRNAs, and miRNAs were jointly profiled to construct an NSCLC diagnostic model based on a multi-RNA expression signature (100). Viewed together, these findings support the incorporation of circulating lncRNAs into multimolecular liquid-biopsy panels rather than their use solely as isolated markers.

A further advantage of liquid biopsy is the feasibility of serial sampling, which enables molecular changes to be tracked during treatment. A plasma EV-derived long-RNA signature has been used to predict response to first-line immunochemotherapy in lung squamous cell carcinoma (101), supporting the potential of EV-RNA profiling for therapeutic stratification. For stress-related lncRNAs, longitudinal studies could compare expression trajectories before treatment, during therapy, and at recurrence, and relate these changes to radiographic response, minimal residual disease, and the emergence of resistance. Nevertheless, exosome isolation, sample processing, RNA quantification, cutoff definition, and analytical platforms remain insufficiently standardized. Large prospective cohorts will be essential before such approaches can be incorporated into routine clinical practice (102, 103).

Spatial and temporal heterogeneity complicates the interpretation of stress-associated lncRNA biomarkers. Multiregion analyses from TRACERx demonstrate substantial transcriptomic diversity within individual NSCLC tumors (143). Accordingly, lncRNA abundance measured in a single biopsy may reflect the sampled cellular composition and local stress state rather than a uniform tumor-wide dependency. Treatment-associated changes may additionally reflect shifts in subclonal abundance, tumor burden, or stromal and immune-cell contributions. These limitations do not invalidate static biomarkers, but they support evaluating their performance across sampling sites, disease stages, and treatment contexts.

For candidate stress-related lncRNAs, longitudinal sampling before treatment, during response, and at progression should be combined, where feasible, with spatial or cell-type-resolved measurements. Circulating lncRNA changes should not be assumed to originate exclusively from malignant cells. Their incremental value should be tested against clinical variables and established molecular measurements using prespecified endpoints and independently validated models.

In addition to expression-based biomarkers, circulating epigenetic and RNA epitranscriptomic signatures have been explored for identifying EGFR mutational status and predicting responses to EGFR-TKIs (104), providing new avenues for patient stratification and treatment-response monitoring that extend beyond conventional lncRNA expression profiling.

It is important to acknowledge that most lncRNA biomarker studies in NSCLC remain retrospective, relying on publicly available databases or single-center cohorts with limited sample sizes. Large-scale, multicenter prospective studies with standardized preanalytical protocols and predefined cutoff values are urgently needed to validate the clinical utility of stress-related lncRNAs and to establish robust reference ranges for routine diagnostic and prognostic applications.

Clinical validation should also distinguish diagnostic discrimination, prognostic association, and prediction of treatment benefit. A survival-associated lncRNA signature is not necessarily predictive of response to a particular therapy. Studies should prespecify the intended clinical use, assay, cutoff, and outcome and evaluate relevant covariates, including age, smoking exposure, stage, histology, driver alterations, performance status, and treatment history. Feature selection and cutoff optimization should be confined to the development dataset, followed by evaluation in independent patient cohorts. Beyond discrimination, calibration and clinical utility should be assessed. Treatment-specific predictive claims ideally require evidence that the biomarker modifies treatment benefit, rather than an association with outcome within a single treated cohort.

Candidate lncRNA biomarkers should be evaluated alongside histology, actionable driver alterations, and treatment-specific clinical variables. In immunotherapy studies, comparisons should include PD-L1 expression and, where relevant, tumor mutational burden, with clear reporting of the assay and cutoff used for each measurement. Association with PD-L1, TMB, or inferred immune infiltration does not establish added predictive value. Such value should be evaluated by comparing a prespecified baseline model with the same model after addition of the lncRNA measurement in an independent cohort. For EGFR-targeted therapy, analyses should specify the EGFR alteration, treatment line, prior TKI exposure, and resistance setting. The proposed role of lncRNAs is therefore an investigational complement to existing stratification approaches rather than a validated basis for treatment selection.

### 4.2 Therapeutic targeting of lncRNAs

Stress-related lncRNAs occupy regulatory positions within multiple tumor-adaptive programs. Suppressing oncogenic lncRNAs or restoring tumor-suppressive lncRNA activity offers an RNA-level strategy for disrupting survival and resistance programs (105, 106). Current approaches include antisense oligonucleotides (ASOs), RNA interference (RNAi), CRISPR-based systems, and interventions directed against lncRNA-encoded micropeptides. (Representative lncRNA targets and their therapeutic implications in NSCLC are summarized in **Table 2**).

**Table 2:** Representative stress-associated lncRNAs and their therapeutic implications in NSCLC.

lncRNA/ncRNA-related target	Associated stress context	Major regulatory mechanism	Therapeutic implication	Evidence level
LINC01278 (85)	EGFR-TKI stress	LINC01278 sponges miR-324-3p to increase ZFX expression	Targeting LINC01278 may resensitize osimertinib-resistant NSCLC	*In vitro* (EGFR-mutant osimertinib-resistant derivatives)
IGF2BP3-associated metabolic axis (129)	EGFR-TKI-induced metabolic stress	IGF2BP3 promotes acquired EGFR inhibitor resistance through metabolic reprogramming and enhanced oxidative phosphorylation	Targeting metabolic dependencies may provide a strategy to resensitize EGFR-TKI-resistant NSCLC. Indirect pathway-level rationale; not a direct lncRNA-targeting intervention	*In vitro* + *In vivo* (preclinical metabolic dependency study)
AFAP1-AS1 (132)	Immunotherapy-associated stress	AFAP1-AS1 enhances JAK2 and TYK2 translation, promoting immune escape and immunotherapy resistance	Potential target for improving immune checkpoint blockade efficacy Preclinical combination with anti-PD-1	*In vitro* + *In vivo*
LINC02178 (120)	Tumor progression and therapeutic adaptation	LINC02178 promotes lung adenocarcinoma progression and represents a target for nanodelivery-based intervention	Preclinical nanodelivery; subcutaneous xenograft	*In vitro* + *In vivo*
Immune-related lncRNA signatures (130, 131)	Tumor immune microenvironment stress	Modulate immune cell interactions and predict immunotherapy response	Potential application in patient stratification and precision immunotherapy	Human observational biomarker study
Exosomal lncRNAs (96, 99, 100)	Tumor microenvironmental stress	Mediate intercellular communication through extracellular vesicle transport	Potential non-invasive biomarkers and therapeutic targets	Human observational biomarker study
PVT1 (29)	Hypoxia & chemotherapy stress	PVT1 sponges miR-140-3p to upregulate ATG5 and enhance autophagy	Combining PVT1 inhibition with cisplatin may sensitize autophagy-mediated resistance	*In vitro* + *In vivo*

Evidence levels reflect the highest reported experimental stage. No lncRNA-targeting agent has entered clinical trials for NSCLC. Combination effects should be interpreted as cooperative unless formal synergy modeling was performed. Subtype and mutational context should be considered when interpreting therapeutic implications. Observational biomarker associations are not evidence of therapeutic efficacy. *In vitro* sensitization does not establish clinical benefit.

The lncRNA-directed interventions discussed in this review provide preclinical evidence for the proposed NSCLC applications rather than established evidence of clinical benefit. Antitumor activity in cultured cells or xenografts does not establish a clinically achievable exposure, an acceptable therapeutic window, or efficacy in patients. The following discussion of targeting modalities should therefore be interpreted as an assessment of experimental approaches and their translational requirements.

ASOs represent one of the most established strategies for targeting lncRNAs. Through sequence-complementary recognition of target RNAs, ASOs induce RNase H-mediated degradation or sterically interfere with RNA processing and molecular interactions (107, 108). ASO-based strategies targeting oncogenic lncRNAs such as MALAT1, PVT1, and NEAT1 have been evaluated in multiple preclinical cancer models (109). Chemical modification, together with delivery platforms such as nanoparticles, polymers, peptide-based carriers, and ligand conjugates, further improve oligonucleotide stability, tissue distribution, and cellular uptake (110, 111).

RNAi acts primarily through siRNA loading into the RNA-induced silencing complex (RISC), followed by degradation of complementary target RNAs, and is generally more effective for cytoplasmic lncRNAs. By contrast, nuclear lncRNAs are often more amenable to ASO-based or transcriptional interventions (112). CRISPR-based technologies further expand the therapeutic toolkit. CRISPR interference (CRISPRi) represses lncRNA transcription through dCas9-KRAB without generating DNA double-strand breaks, whereas conventional Cas9 alters lncRNA expression through editing of promoters or genomic loci (113). Cas13 directly targets RNA and enables transcript-level knockdown without modifying genomic DNA; however, guide-RNA specificity and the potential for collateral RNA cleavage remain important considerations (114).

Through this mechanism, the selection of targeting modality should be guided by lncRNA subcellular localization: nuclear-enriched lncRNAs are generally more amenable to ASO-mediated degradation or CRISPRi-based transcriptional repression, whereas cytoplasmic lncRNAs can be effectively targeted by RNAi to harness the RISC machinery.

Beyond the four canonical RNA-level regulatory modes discussed above, an emerging layer of lncRNA-mediated stress adaptation involves lncRNA-encoded micropeptides. Although historically overlooked due to their small size and non-coding annotation, these peptides have been increasingly recognized as direct effectors of metabolic reprogramming, redox homeostasis, DNA-damage response, and therapeutic resistance in cancer (115). This represents an emerging paradigm in which lncRNA loci possess dual functionality, exerting effects through both the RNA transcript and the encoded micropeptide, which significantly expands the therapeutic target space. A well-characterized instance in NSCLC is ATMLP, a mitochondria-targeted micropeptide encoded by AFAP1-AS1 that confers resistance to ionizing radiation. This phenotype is underpinned by three concurrent activities: ATMLP quenches irradiation-elicited ROS, reinforces AKT-mediated survival signaling, and enhances lipid-droplet storage (87). Depending on the dominant effector mechanism, therapeutic strategies may target the RNA transcript itself, its translation, or the interaction interface between the encoded micropeptide and its protein partners.

The principal technical barriers to nucleic-acid therapeutics include stability, tissue distribution, cellular uptake, and intracellular release (116–119). The extent to which delivery systems are required differs across nucleic-acid modalities. Some chemically modified ASOs achieve tissue uptake without a dedicated nanocarrier, whereas double-stranded siRNAs are generally more dependent on specialized delivery platforms (117, 118). Lipid nanoparticles are among the most important RNA-delivery systems currently available, although their application to solid tumors remains constrained by stromal penetration, nonspecific biodistribution, and intratumoral heterogeneity.

Direct preclinical evidence for lncRNA-targeted nanodelivery has now emerged in LUAD. A nanoparticle formulation carrying siRNA against LINC02178 reduced LINC02178 expression, suppressed LUAD cell proliferation, promoted apoptosis, and decreased tumor burden in a subcutaneous xenograft model (120). This study establishes a continuous preclinical trajectory from lncRNA target selection to siRNA silencing, nanocarrier-mediated delivery, and *in vivo* antitumor activity, illustrating the therapeutic potential of lncRNAs with well-defined oncogenic functions and tractable delivery properties.

Lung cancer also offers an anatomical opportunity for local pulmonary delivery. Inhaled RNA formulations act directly within the respiratory tract and lung tissue while limiting, to some extent, systemic exposure. Preclinical proof-of-concept has been reported for inhaled siRNA nanoplatforms targeting KRAS-mutant NSCLC and for RNA delivery in orthotopic lung tumor models (121). Coupling pulmonary delivery with stress-related lncRNA targets may in turn increase local drug exposure within lung lesions and provide a practical route for repeated administration.

### 4.3 Combination strategies based on lncRNA targeting

lncRNA-mediated therapeutic resistance encompasses autophagy, metabolic reprogramming, DNA repair, drug transport, epithelial-mesenchymal transition (EMT), and programmed cell-death pathways (122–124). A major therapeutic rationale for targeting lncRNAs is to weaken the adaptive buffering capacity established under sustained treatment pressure and thereby increase tumor sensitivity to existing therapies.

During chemotherapy, lncRNAs modulate responses to cisplatin and taxanes through autophagy, evasion of apoptosis, and metabolic adaptation. Hypoxia-induced PVT1 enhances autophagy through the miR-140-3p/ATG5 axis and promotes cisplatin resistance (29), whereas H19 supports cisplatin resistance by maintaining mitophagy (83). In addition, FEZF1-AS1-driven glutamine metabolic reprogramming and the involvement of MNX1-AS1 in ferroptotic and paclitaxel responses indicate that chemoresistance can be shaped simultaneously by metabolic, redox, and cell-death programs (125). Targeting specific resistance-promoting lncRNAs restores drug sensitivity in preclinical settings (123). Because processes such as autophagy are strongly dependent on tumor context and treatment stage (126), rational combination strategies should be guided by the dominant resistance mechanism rather than by indiscriminate multi-target intervention.

EGFR-TKI resistance exhibits an even more pronounced network architecture. Multiple lncRNAs sustain cell survival after EGFR inhibition through glutamine metabolism, autophagy, EMT, apoptosis regulation, and bypass signaling via PI3K/AKT, MAPK, and JAK/STAT pathways (127, 128). The lncRNAs discussed in Chapter 3, including MYLK-AS1, HAS2-AS1, LINC00969, and LINC01278, respectively connect metabolic reprogramming, epigenetic regulation, cell-death control, and ceRNA networks, illustrating how distinct adaptive nodes converge functionally under targeted-therapy pressure. At a broader level of compensatory dependency, inhibition of oxidative phosphorylation in combination with gefitinib has enhanced antitumor activity in cell-derived xenograft (CDX), patient-derived xenograft (PDX), and patient-derived organoid (PDO) models (129). These findings provide a functional rationale for identifying metabolically dependent lncRNAs and developing mechanism-guided combinations of lncRNA targeting with EGFR-TKIs.

In immunotherapy, lncRNAs modulate PD-1/PD-L1 signaling, CD8+ T-cell activity, regulatory T-cell (Treg) states, and macrophage polarization, thereby contributing to an immunosuppressive tumor microenvironment (91, 130). Immune-related lncRNA signatures have also been associated with tumor mutational burden (TMB), immune infiltration, and immune-checkpoint expression and assist in candidate patient stratification (131). AFAP1-AS1 provides a particularly well-defined mechanistic and therapeutic example. By interacting with EIF4A1, AFAP1-AS1 enhances JAK2/TYK2 translation and activates STAT1, leading to increased PD-L1 and IDO1 expression and impaired CD8+ T-cell cytotoxicity. Targeting this signaling axis in combination with anti-PD-1 therapy enhanced antitumor activity in preclinical models (see **Table 2**) (132). These observations support further preclinical investigation of selected lncRNAs as indicators of immune state and candidate intervention points for immune modulation.

Radiotherapy similarly imposes substantial oxidative, genotoxic, and metabolic stress that can be buffered by lncRNA-associated programs. The mitochondrial micropeptide ATMLP, encoded by AFAP1-AS1, reduces ionizing-radiation-induced ROS accumulation while promoting AKT signaling and lipid-droplet formation, thereby increasing radioresistance in NSCLC (87). In parallel, DNA repair-related lncRNAs influence responses to radiotherapy and other genotoxic therapies by modulating chromatin accessibility and DNA-damage repair programs. These observations highlight the potential of lncRNAs, their encoded micropeptides, and downstream stress-adaptation pathways as targets for radiosensitization.

The rationale for combining lncRNA perturbation with established treatments is to test whether disruption of a defined adaptive dependency improves treatment response. Greater activity than either intervention alone does not, by itself, establish pharmacological synergy. A synergy claim requires an appropriate dose–response design, a specified reference model such as Bliss independence, Loewe additivity, or a justified combination-index approach, and assessment of experimental variability (144). The inference is specific to the tested dose range, schedule, endpoint, and experimental model.

Where formal interaction analysis is unavailable, findings should be described as sensitization or an enhanced response in the tested preclinical system. Genetic depletion of an lncRNA should also be distinguished from administration of a clinically deliverable RNA-targeting agent. For example, LINC01278 depletion supports an *in vitro* resensitization hypothesis in selected osimertinib-resistant models (85), whereas the IGF2BP3-associated metabolic study provides an indirect rationale for investigating adaptive dependencies rather than direct validation of lncRNA-targeted combination therapy (129). Clinical translation will require product-level delivery, target-engagement and safety studies, followed by prospective evaluation in appropriately selected patients.

### 4.4 Barriers to clinical translation

The clinical translation of stress-related lncRNAs is constrained by interrelated challenges in delivery, safety, clinical validation, analytical standardization, and product development.

Before reaching their targets, nucleic-acid therapeutics must overcome four barriers: *in vivo* degradation, tissue distribution, tumor penetration, and intracellular release (116–119). Although local pulmonary administration offers organ-specific advantages, mucus barriers, mucociliary clearance, macrophage uptake, and the safety of repeated dosing remain unresolved (133). Lipid nanoparticle-based delivery can improve the stability and tissue delivery of RNA therapeutics, although its application to solid tumors remains limited by poor stromal penetration and nonspecific biodistribution (119).

These barriers also require a distinction between tissue accumulation and productive target engagement. Detection of an oligonucleotide in lung tissue does not necessarily demonstrate delivery to the relevant malignant, stromal, or immune-cell population, endosomal escape, or access to the intracellular compartment containing the target lncRNA. Safety assessment should consider sequence-dependent off-target hybridization, chemistry- or carrier-associated toxicity, innate immune activation, and on-target effects in normal tissues (106, 116–119). Independent targeting sequences and appropriate rescue experiments can strengthen the attribution of an observed phenotype to the intended lncRNA, but they do not replace dedicated pharmacokinetic, pharmacodynamic, and toxicological evaluation.

Clinical validation further requires standardized pipelines and companion diagnostic development. Candidate lncRNA targets should undergo stepwise validation from three-dimensional cultures and organoids to *in vivo* models before entering clinical development, with particular attention to assay reproducibility across sample types and processing protocols (134, 135). Robust clinical implementation of exosomal lncRNA biomarkers will require standardized isolation and analytical procedures, predefined expression cutoffs, and prospective multicenter validation to ensure reproducible patient stratification (102). Given the heterogeneity of NSCLC stress-adaptation programs, future patient-stratification frameworks should move beyond single-lncRNA thresholds and incorporate lncRNA profiles together with molecular and microenvironmental context.

Analytical variability represents an additional obstacle. Differences in sample processing, isolation, quantification, and normalization of circulating lncRNAs compromise reproducibility across studies (100, 103). Standardization should therefore cover specimen type, collection conditions, processing delay, hemolysis assessment, storage, freeze–thaw exposure, RNA extraction, and reverse transcription. Assays should distinguish relevant transcript isoforms and report analytical specificity, amplification efficiency, detection limits, and quantification limits. Reference transcripts should be validated in the specimen and stress condition under investigation rather than assumed to remain stable during metabolic or therapeutic stress.

RT-qPCR reporting should follow MIQE 2.0 principles (145), while extracellular-vesicle studies should incorporate MISEV2023 recommendations for separation, characterization, and reporting (146). External spike-ins can help monitor technical recovery but do not replace biological normalization. Target validation, analytical validation, and patient-stratification development should be coordinated from the outset to improve comparability across studies and support subsequent clinical evaluation.

Regulatory development requires a clearly defined therapeutic product, reproducible manufacturing, and an appropriate benefit–risk assessment. Relevant guidance for oligonucleotide therapeutics already exists, including the FDA guidance on clinical pharmacology considerations and the EMA draft guideline on development and manufacture. The translational challenge is therefore to apply these frameworks to a specific lncRNA-directed agent and delivery platform, including characterization of identity, purity, potency, stability, batch consistency, exposure, and target engagement.

Commercial development should consider manufacturing scalability, formulation complexity, biomarker-defined patient recruitment, and the resources required for clinical validation. Intellectual-property assessment should distinguish claims concerning naturally occurring RNA sequences from those concerning engineered agents, formulations, or therapeutic applications. Patentability and freedom to operate require case-specific evaluation. These considerations represent potential development constraints rather than evidence that lncRNA therapeutics categorically lack patent protection or an applicable regulatory framework.

## 5 Current limitations and future perspectives

### 5.1 Current research gaps

Three fundamental gaps constrain current understanding of lncRNA-mediated stress adaptation in NSCLC.

First, experimental models remain inadequate. Most studies rely on two-dimensional cultures and single-stressor paradigms, which cannot reproduce the simultaneous nutrient deprivation, hypoxia, acidification, redox imbalance, immune interactions, and therapeutic pressure encountered in solid tumors (135). Although organoids and patient-derived xenografts preserve selected features of parental tumors more faithfully, neither fully recapitulates immune, vascular, or systemic environments. The limited cross-species sequence conservation of many lncRNAs further constrains the functional validation of lncRNAs in animal models, as phenotypes observed in murine systems may not translate to human lncRNA orthologs (98).

Model selection should therefore be matched to the biological question. Human tumor xenografts can support investigation of tumor-cell dependencies, but an immunodeficient host cannot reproduce intact human antitumor immunity. Immune-containing patient-derived lung cancer organoids provide an additional experimental approach for examining local treatment responses (147), although they do not reproduce systemic exposure or organism-level toxicity. For human-specific lncRNAs, conservation of sequence, expression, interacting partners, and function should be evaluated separately. Complementary testing in human tumor models, relevant immune or stromal co-cultures, and normal human tissue models would help address limitations that cannot be resolved by a single experimental system.

Second, upstream regulatory mechanisms remain understudied. The field has focused far more extensively on how lncRNAs regulate metabolism or cell death than on how environmental stress first alters lncRNA expression or function (92). This gap is particularly evident in oxidative stress, where direct evidence tethering ROS to lncRNA transcription, stability, processing, or subcellular localization remains limited. Most studies describe “stress changes lncRNA expression” without dissecting the intervening transcriptional, post-transcriptional, or epigenetic mechanisms.

Third, functional attribution remains technically challenging. Genomic deletion simultaneously perturbs neighboring regulatory elements and the transcriptional process itself, whereas RNAi and ASOs mainly act on mature transcripts. Different perturbation strategies in effect yield divergent phenotypes (134). Compounding this, the persistent emphasis on one-lncRNA/one-pathway models means that relatively few studies simultaneously examine two or more stress inputs or how lncRNA functions switch or become compensated under changing conditions. This reductionist design limits a systems-level understanding of long-term adaptation in NSCLC.

### 5.2 Future research directions

A priority for future research is to construct single-cell and spatial maps of stress-related lncRNAs. Single-cell transcriptomics resolve cell type-specific lncRNAs that are obscured in bulk RNA-seq and delineate their relationships with metabolism, EMT, immune states, and treatment response. Cross-cancer studies have already demonstrated the ability of this approach to reveal intratumoral lncRNA heterogeneity (136). Integrating spatial transcriptomics could further determine whether candidate lncRNAs are preferentially localized to hypoxic regions, immune-infiltrated niches, necrotic margins, or residual tumor areas after therapy.

Multi-omics integration and interpretable machine learning promise to help identify key nodes shared across stress programs. Cross-cancer studies have combined bulk transcriptomics, single-cell data, immune features, and machine learning to construct stress-related lncRNA models (137). Future NSCLC studies could incorporate RNA modifications, chromatin accessibility, metabolomics, and proteomics so that predictive frameworks move beyond risk classification toward mechanistic questions of which stress activates a given lncRNA and what adaptive consequence follows.

Experimental models should likewise progress from two-dimensional single-stressor systems toward three-dimensional compound-stress platforms. Tumor spheroids and patient-derived organoids retain selected aspects of three-dimensional architecture and drug response, and lung cancer studies have already examined multiple lncRNAs in 3D models in relation to tumor growth or therapeutic response (138). Future systems could combine hypoxia, nutrient restriction, lactate-driven acidification, oxidative stress, and drug exposure, together with co-culture of cancer-associated fibroblasts (CAFs), macrophages, or T cells, to evaluate lncRNA function under more physiologically relevant multistress conditions.

At the same time, stricter causal-validation frameworks should be established using orthogonal approaches such as RNAi, ASOs, CRISPRi, genomic editing, and RNA rescue (134). With respect to delivery, inhaled RNA nanoplatforms have already achieved preclinical proof-of-concept in KRAS-mutant NSCLC (121) and could be adapted to stress-related lncRNA targets. Biomarker development should also move beyond retrospective database analyses toward multicenter prospective validation, with standardized sampling and analytical procedures and with emphasis on compact circulating-lncRNA panels that combine high stability with high specificity (100, 103).

Finally, lncRNA-encoded micropeptides represent an underexplored layer of stress adaptation; future studies should systematically identify functional micropeptides encoded by stress-related lncRNAs and evaluate their therapeutic potential, as dual-function lncRNA loci may offer novel target interfaces beyond conventional RNA-targeting strategies.

Ultimately, mechanistic studies, drug development, and biomarker research should be integrated into a continuous translational framework. Liquid biopsy or tissue-based omics could first be used to define the dominant stress state in an individual tumor, followed by identification of the corresponding lncRNA dependency, selection of an appropriate targeted or combination regimen, and serial circulating-RNA measurements to monitor therapeutic response and the evolution of resistance (**Table 3**).

**Table 3:** Current challenges and future directions for clinical translation of stress-associated lncRNAs in NSCLC.

Current challenge	Key limitation	Future direction	References
Incomplete functional annotation of lncRNAs	Many stress-responsive lncRNAs remain poorly characterized and their biological functions require further validation	Combining reverse genetics, CRISPR-based screening and multi-omics approaches	(105, 134)
Dynamic tumor stress heterogeneity	Tumor cells experience simultaneous metabolic, hypoxic, oxidative and immune stresses that are difficult to reproduce in conventional models	Applying single-cell sequencing, spatial transcriptomics and advanced tumor models	(92, 136)
Limited clinical validation of biomarkers	Most candidate lncRNA biomarkers require larger cohorts and standardized detection methods	Establishing multicenter validation studies and clinical assay platforms	(99, 100)
Delivery barriers for RNA-based therapeutics	Poor stability, insufficient tumor targeting and biological barriers restrict clinical translation	Developing nanoparticles, extracellular vesicles and lung-targeted delivery systems	(107, 117, 121, 133)
Insufficient prediction models for treatment response	Tumor heterogeneity limits accurate prediction of therapeutic sensitivity	Integrating lncRNA signatures with immune profiling and multi-omics analysis	(127, 131, 137)
Subtype and mutation specificity	Many mechanisms are studied in single models and generalized to NSCLC	Validate in LUAD and LUSC and in EGFR, KRAS, STK11/LKB1, KEAP1/NRF2-defined models	(92, 127, 131)
Integration with established biomarkers	PD-L1 and TMB are often not incorporated into lncRNA studies	Combine lncRNA signatures with PD-L1, TMB, and multi-omics	(99, 100, 131)

This table summarizes translational challenges identified from the reviewed literature and proposed directions. It does not imply that any lncRNA-targeting strategy has entered clinical practice for NSCLC.

## 6 Conclusions

This review has examined lncRNA-mediated multistress adaptation in NSCLC through a network-based framework that positions lncRNAs as stratified regulatory nodes within a cross-stress compensatory network. Rather than cataloging individual lncRNA–target axes, this framework emphasizes how four stress dimensions—metabolic reprogramming, redox regulation and ferroptosis, the hypoxic/acidic/immune microenvironment, and therapeutic resistance—reciprocally feed into one another. Within this network, lncRNAs occupy distinct positions along the stress-input-to-adaptive-output continuum.

Mechanistically, lncRNA activity extends well beyond ceRNA competition. lncRNAs regulate metabolism, antioxidant defenses, autophagy, DNA repair, and immune evasion through RNA-protein interactions, chromatin and RNA modifications, changes in subcellular localization, extracellular-vesicle-mediated communication, and micropeptide coding. The same lncRNA also engages distinct mechanisms in different cellular compartments, allowing a single transcript to connect otherwise separate functional modules.

lncRNAs also differ in their positions within the stress-response hierarchy. A subset displays a relatively continuous chain from environmental stimulus to upstream regulation, altered lncRNA abundance or activity, and an adaptive phenotype, placing these molecules closer to the stress-input layer. Many others function primarily as downstream effectors that sustain or amplify metabolic adaptation, ferroptosis regulation, immune modulation, and therapeutic resistance. Distinguishing these functional levels avoids treating every lncRNA associated with a stress phenotype as an environmental sensor.

At the systems level, NSCLC adaptation does not follow a simple unidirectional cascade. Metabolic reprogramming alters lactate production, ROS levels, and reducing equivalents, thereby influencing ferroptosis sensitivity and epigenetic regulation. Hypoxia further reshapes both metabolic and immune states, whereas therapeutic pressure reutilizes pre-existing autophagic, DNA-repair, metabolic, and antioxidant programs. Taken together, these interactions support a model in which long-term tumor survival emerges from multiple stress inputs, reciprocal feedback, and compensatory adaptation.

This framework also suggests new avenues for clinical translation. Circulating lncRNAs and multi-RNA liquid-biopsy models may support patient stratification and state monitoring (99, 100), whereas ASOs, siRNAs, CRISPR-based systems, and nanodelivery platforms provide a technical basis for functional intervention. An siRNA nanoplatform targeting LINC02178 has already shown antitumor activity in preclinical LUAD models (120), while studies of AFAP1-AS1 further indicate that lncRNA-associated immunosuppressive programs can be therapeutically coupled to immune-checkpoint blockade (132). Stress-related lncRNAs warrant further evaluation as candidate biomarkers and therapeutic targets.

The field nevertheless remains in transition from mechanistic discovery to clinical translation. Incomplete compound-stress models, difficulties in assigning lncRNA function, limited efficiency of RNA delivery, and insufficient standardization of clinical assays continue to restrict translational progress. Future studies that combine single-cell and spatial multi-omics to define cellular origin and stress context, organoid and compound-stress systems to improve functional fidelity, and lung-directed delivery with prospective clinical cohorts could establish a more complete pipeline from mechanistic discovery and target validation to patient stratification and therapeutic monitoring.

In light of this, the objective of future lncRNA research should not be to identify a single ‘master’ molecule applicable to all patients with NSCLC. A more informative goal is to define the key dependencies that determine tumor adaptation within specific genetic backgrounds, microenvironmental conditions, and treatment contexts. Integrating patient-specific stress states with lncRNA dependency and therapeutic selection may provide a more systematic framework for understanding NSCLC heterogeneity, the evolution of resistance, and precision combination therapy. The proposed framework organizes experimentally reported mechanisms and generates testable hypotheses about context-dependent lncRNA vulnerabilities. It does not establish a clinically validated treatment-selection algorithm. Progress toward clinical use will require reproducible target attribution, histological and genomic stratification, effective delivery, and prospective demonstration of biomarker or therapeutic utility.
